# Beauty, elegance, grace, and sexiness compared

**DOI:** 10.1371/journal.pone.0218728

**Published:** 2019-06-21

**Authors:** Winfried Menninghaus, Valentin Wagner, Vanessa Kegel, Christine A. Knoop, Wolff Schlotz

**Affiliations:** Max Planck Institute for Empirical Aesthetics, Frankfurt am Main, Germany; University of Melbourne, AUSTRALIA

## Abstract

Beauty is the single most frequently and most broadly used aesthetic virtue term. The present study aimed at providing higher conceptual resolution to the broader notion of beauty by comparing it with three closely related aesthetically evaluative concepts which are likewise lexicalized across many languages: elegance, grace(fulness), and sexiness. We administered a variety of questionnaires that targeted perceptual qualia, cognitive and affective evaluations, as well as specific object properties that are associated with beauty, elegance, grace, and sexiness in personal looks, movements, objects of design, and other domains. This allowed us to reveal distinct and highly nuanced profiles of how a beautiful, elegant, graceful, and sexy appearance is subjectively perceived. As aesthetics is all about nuances, the fine-grained conceptual analysis of the four target concepts of our study provides crucial distinctions for future research.

## Introduction

Both in lay concepts of aesthetic perception and evaluation [1–3] and in empirical research, beauty is by far the single most important aesthetic virtue term. To be sure, already 18th century aesthetics knew of several other target virtues, such as the sublime ([4–6]; see also [7–9]) and the interesting ([10,11]; see also [12–14]). Today, the overall picture is far more diversified (for a survey of several dozens of relevant terms, see [2] and [15]). Still, beauty ratings remain the prime currency of empirical studies in aesthetics. Supporting this tendency, theoretical accounts of aesthetic appeal and concomitant pleasure and liking––such as the *processing fluency* hypothesis cf. [16–21]––also show a bias towards explaining attributions of beauty rather than of the sublime or of other aesthetic virtues.

The present article does not challenge this primacy of beauty. Rather, it is aimed at an *inner diversification* of the beauty pole of aesthetics. It is distinctive of the concept of beauty that is used in a very broad way, at least so in the Western tradition since Greek antiquity. On the one hand, it encompasses the sexual beauty of bodily looks and *natural* beauty in general (landscapes, animals, etc.); on the other hand, beauty is also attributed to a great variety of *cultural* artefacts, including classical artworks and numerous objects of design. In the present study, we focus on three concepts each of which we expected to show great semantic overlap with the broader concept of beauty, while at the same time distinctly capturing special facets of beauty on the *nature-culture* continuum.

1. For the cultural beauty pole, we selected “elegance.” In Latin, “elegantia” meant refinement, tasteful correctness, and decorousness, with a specific focus on carefully choosing––lat. *eligere*, to elect––one’s words [22]. Starting in the 14^th^ century, the concept has increasingly come to be applied to multiple domains, including refined personal looks, many objects of design (evening wear, jewelry, hairstyles, furniture, cars––specifically, cabriolets and limousines––, yachts, villas, restaurants, bridges, skyscrapers, etc.), movements, verbal articulation and argumentation, and to unexpectedly smooth solutions to difficult cognitive or technical problems (see S1 Text). Compared to beauty, elegance is far less frequently and far more selectively applied to natural objects, with tigers, leopards and other cats being among the exceptions (not least by virtue of their elegance in movement).
2. For the natural (sexual) beauty pole, we selected the modern term “sexy.” To be sure, this term is not exclusively applied to natural bodily looks, but also to clothing and self-styling and hence to cultural ways of enhancing or highlighting physical sexual attractiveness. The term “sexy” has no tradition in modern philosophical aesthetics, as many, though by no means all authors (see, for instance, Edmund Burke, [4], p. 115) programmatically tried to purge aesthetics and even the theory of beauty of all reminders of sexual desire and biological functions. At the same time, a sexual dimension of beauty attributions to individuals plays an important role in the cultural record from antiquity through modernity, and Charles Darwin’s account of competitive beauty displays throughout nature and human cultures lends much credit to this rich tradition [23].
3. As a third term closely related to beauty, we included the concept of “grace.” Schiller defined grace (German *Anmut*) as “beauty in motion” ([24]; see also Burke [4], p. 119 and [25]), whereas Lord Kames defined it as “elegance in movement” ([26], p. 364). Today, too, grace is nearly exclusively attributed to the movements of people (and some animals), but barely ever to inanimate objects. Regarding the nature-culture-continuum of beauty, grace, for all its overlap with elegance and hence cultural sophistication, appears to lean more towards the natural pole in that graceful movements can well be found in young children and animals and hence are less dependent on cultural sophistication than typical forms of elegance in movement, such as in ballet and other forms of dance. Still, in accordance with the classical definitions, we expected that grace should show far greater overlap with elegance and beauty than with sexiness.

Remarkably, across all major Germanic, Romance and Slavic languages, the Finno-Ugric languages, many Asian languages (Chinese, Japanese, Korean), and potentially beyond, words for “elegance” are lexicalized in phonologically convergent form, i.e., based on the Latin word “elegantia.” For “grace” and “sexy,” a similar adoption of the Latin words “gratia” and “sexus” applies across many languages, albeit not to the same degree. The successful migration of these terms into many other languages suggests that they capture special facets of aesthetic appeal that are salient, in however culturally varying form, across centuries and cultures.

Our study is the first to comparatively analyze the four categories under scrutiny––beauty, elegance, grace, and sexiness––for their distinct perceptual, cognitive and affective implications that together make up their meaning. Specifically, it is aimed at elucidating, with high granularity, the mental constructs which individuals have acquired regarding beauty, elegance, grace, and sexiness. Linguistic concepts are reflective of mental constructs largely shared across linguistic communities, in some cases also across different languages and cultures. Our study tapped into these mental constructs using a variety of methods, including free associations, questionnaires and semantic differentials. We did not present any beautiful, elegant, graceful, and/or sexy stimuli in rating tasks. Rather, we exclusively targeted the very constructs that guide individuals when they end up perceiving and categorizing specific phenomena as beautiful, elegant, graceful, or sexy.

### The two studies

For efforts aimed at contrasting hypothetical sub-types of a broader class of phenomena, the general category is the unmarked class. A focus on bringing out differences between our four categories may therefore be better served by placing a primary focus on one of the more circumscribed categories. Following this reasoning, our first study was primarily devoted to investigating characteristics of elegance. It opens its scope to also include beauty and sexiness only in the part which addresses the factor of age in attributions of beautiful, elegant and sexy personal looks.

In the absence of a full-blown theory of elegance––in fact, of any *theory* of elegance––, we resorted to well-established methods of investigating uncharted phenomena and concepts, such as free associations and semantic differentials (for all details, see the Method sections of Studies 1 and 2). We designed a variety of open-format questions as well as scales and questionnaires aimed at elucidating which cognitive, emotional, and social characteristics are associated with elegant individuals and which features of movement and verbal performance are considered elegant. In this context, we also collected the very first data sets that directly compare perceived beauty, elegance, and sexiness in personal appearance.

Study 2 extended this comparison to a broad range of cognitive, affective, and social implications associated with all four target categories of our study. Informed by the primarily elegance-related results of Study 1 as well as by theoretical anticipations, we collected, in a between-subject design, ratings for beauty, elegance, grace, and sexiness, respectively, on 42 semantic differentials (i.e., polar adjective pairs). Osgood and colleagues [27] introduced semantic differentials as a method to analyze with high conceptual resolution even minor differences of target categories on subjectively perceived qualitative dimensions. We adopted this method for our purposes.

## Study 1

The first part of Study 1 consisted of a free association task: participants were asked to write down words that they associate with elegance. Association tasks of this type have been shown to provide access to conceptual affinities informing the meaning of a specific concept [28,29]. The subsequent blocks of questions targeted person-related characteristics dependent on an overall elegant personal appearance and descriptors of elegant physical build and gait. As elegant diction is historically *the* classical domain of elegance, we also investigated to what extent contemporary individuals have a clear understanding of this type of elegance, too. To this end, we presented them with attributes that are often associated with verbal elegance.

Going beyond elegance only, we also asked our participants to indicate whether or not it is likely in their view that women and men, respectively, are perceived as beautiful, elegant, and/or sexy in predefined age ranges.

### Methods

#### Participants

One hundred fifty individuals participated in this study (115 female, 34 male, 1 undisclosed; mean age in years *M* = 30.1, *SD* = 14.4, median = 24, min = 18, max = 77; for a histogram of the age distribution, see S2 Text). They were mostly recruited via the general study participant database of the Max Planck Institute for Empirical Aesthetics (which by the time the study was performed comprised some 1.500 participants covering a broad range in age, educational profiles, professions, etc.).

Most of the participants (77%) reported a monthly income of up to 1,500 EUR. 34% held a university degree or similar qualification; of the remaining participants, 56% held a secondary school diploma (Abitur). The age distribution and the data on the highest degree of education together indicate that the greater part of the sample had attended or were currently attending university.

One hundred forty-six participants were native speakers of German, 20 of them bilingual. We report the results for all 150 participants, as excluding the four non-native speakers did not change the results. (For further details on the sample, see S2 Text.)

#### Ethics statement

Study 1 was conducted in full accordance with the World Medical Association’s Declaration of Helsinki and the Ethical Guidelines of the German Association of Psychologists (DGPs). All participants gave their written informed consent and received EUR 5 as compensation for their participation. All procedures of the study were approved by the Ethics Council of the Max Planck Society.

#### Questionnaire

The questionnaire (which is available at the Open Science Framework, https://osf.io/uqga9/) was presented on a computer in a lab room. In the free association task, participants were asked to write down between one and twenty words they associate with elegance.

Subsequently, they were asked to indicate on 7-point Likert scales how strongly they associate elegant individuals with several cognitive, emotional, and social characteristics, and to what degree they consider specific features as distinctive of elegance in gait and diction. Again, in the absence of available scales, we selected our rating items in a lengthy process that was informed by our intuitive understanding of elegance, lexicon entries, and discussion among the authors. We also consulted research on personality attributions dependent on physical attractiveness [30–32].

Additionally, we collected data on the physical build, height, and leg length participants considered prototypical for an elegant personal appearance. For physical build, participants could indicate whether attributes such as “slender,” “corpulent”, “athletic,” etc. are compatible with an elegant appearance. For both height and leg length, participants could choose between three degrees (tall/long, medium, small/short).

In a next step, we asked our participants to indicate categorically for each of seven predefined age ranges whether or not they consider it likely that women and men, respectively, are perceived as beautiful, elegant, and/or sexy in these age ranges. We limited the lowest age range to the four years from age 16 to 19 in order to rule out that kids younger than sixteen years of age would need to be projected onto the mental template of sexual appeal. Above this age, we collected data for all decades from age 20 to 90. We did not ask *how great a percentage* of men and women of these age ranges they perceive as beautiful, elegant, and/or sexy, because we expected that this would be a fairly demanding question that could prevent our participants from making a quick, spontaneous choice. Still, as participants responded to the same task for all age ranges, we expected that the data should well be informative regarding *relative differences* in subjective perceptions of beautiful, elegant, and sexy personal looks dependent on age.

Furthermore, participants were asked (1) to list public figures they find elegant, (2) to rate several experimenter-selected classes of objects regarding a) how frequently individual items in these classes are elegant, and b) the degree of elegance these objects can reach in their view, and (3) indicate their own personal habits and preferences regarding elegant clothing. The results regarding these questions are reported in the Supporting Information (see S4 and S5 Tables and S2 Text).

### Results and discussion

The data and analysis scripts for Study 1 and 2 are available at Open Science Framework (https://osf.io/uqga9/).

#### Free association data

On average, participants wrote down 11.3 associations (median = 10, *SD* = 4.96, min = 2, max = 20, with 24 participants producing the allowed maximum of 20 associations). We preprocessed the data by correcting spelling mistakes and by subsuming all nouns and adjectives of the same word stem (such as “beauty” and “beautiful”) under one category only. All in all, 1,699 associations and 816 words (637 after categorizing) were generated.

For the following analysis, we retained only the 43 words that were mentioned by at least 5% of the participants. Frequencies, mean list rank, and the Cognitive Salience index for these 43 predominant associations are reported in Table 1. The Cognitive Salience index reflects the absolute Frequency of the terms mentioned (= *F*) divided by the product of the Number of participants (= *N*) and the Mean List Rank of the terms *(MLR)*: CSI = F/(N * MLR). The CSI is bounded between 0 and 1, with higher values reflecting higher salience of words for a conceptual domain [33].

**Table 1:** Words most frequently associated with elegance, ranked by their cognitive salience index (CSI).

English	German	freq.	rel.freq.	MLR	CSI
beauty	Schönheit	76	50.7%	3.47	.146
grace	Anmut	27	18.0%	2.89	.062
aesthetics	Ästhetik	43	28.7%	5.09	.056
style	Stil	30	20.0%	4.87	.041
svelte	grazil	20	13.3%	3.95	.034
clothing	Kleidung	22	14.7%	4.55	.032
fashion	Mode	24	16.0%	5.29	.030
dance	Tanz	24	16.0%	6.08	.026
simple	schlicht	16	10.7%	4.13	.026
chic	schick	20	13.3%	5.60	.024
lightness	Leichtigkeit	15	10.0%	4.73	.021
movement	Bewegung	18	12.0%	5.72	.021
refined	edel	15	10.0%	5.40	.019
women	Frauen	13	8.7%	4.77	.018
dress	Kleid	13	8.7%	5.08	.017
looks	Aussehen	13	8.7%	5.38	.016
classic	klassisch	13	8.7%	5.46	.016
supple	geschmeidig	13	8.7%	5.62	.015
self-confidence	Selbstbewusstsein	16	10.7%	6.94	.015
lady	Dame	14	9.3%	6.07	.015
feminine	weiblich	12	8.0%	5.50	.015
posture	Haltung	14	9.3%	6.57	.014
charisma	Ausstrahlung	14	9.3%	6.64	.014
evening gown	Abendkleid	11	7.3%	5.27	.014
black	schwarz	11	7.3%	5.36	.014
art	Kunst	15	10.0%	7.33	.014
expensive	teuer	13	8.7%	6.38	.014
delicate/fine	fein	13	8.7%	6.69	.013
calm	Ruhe	14	9.3%	7.43	.013
suit	Anzug	12	8.0%	6.67	.012
sublime	erhaben	9	6.0%	5.11	.012
stylish	stilvoll	11	7.3%	6.45	.011
fluent	fließend	10	6.7%	6.00	.011
harmonious	harmonisch	10	6.7%	6.30	.011
ballet	Ballett	10	6.7%	7.10	.009
glamour/splendor	Glanz	10	6.7%	7.60	.009
gait	Gang	8	5.3%	6.13	.009
wealth	Reichtum	11	7.3%	8.73	.008
music	Musik	9	6.0%	7.33	.008
appearance	Erscheinungsbild	10	6.7%	8.50	.008
poetry	Poesie	8	5.3%	6.88	.008
gentle	sanft	8	5.3%	7.00	.008
distinguished	vornehm	8	5.3%	7.38	.007

*Note*. freq. = frequency; rel. freq. = relative frequency; MLR = mean list rank; CSI = cognitive salience index.

Highlighting the close affinity of three of our target concepts, participants associated *elegance* most frequently and most saliently with *beauty* and *grace*. In contrast, our fourth target concept, *sexy*, was not listed at all. *Aesthetics*, *style*, *svelte*, *clothing*, and *fashion* followed next. Exploratory analyses of the data revealed a fine-grained, complex network of associations (see S3 Text and S1, S2 and S3 Figs). As these analyses yielded no clear-cut clusters or dimensions, we here base our discussion on semantic grounds. We distinguish four groups of word associations:

#### (1) General perceptual qualities and aesthetic appeal dimensions of elegance

Entries include impressions of *lightness/delicacy*, *fluency* (*supple*, *fluent*), *harmony*, *calm* and *exquisiteness* (*glamour/splendor*, *finesse*, *classic*, *charisma*). For all these extraordinary virtues, elegance is still associated with *simplicity*. Three of these major characteristics of elegance––namely, *fluency*, *lightness*, and *simplicity*––represent key concepts, or synonyms thereof, of the *processing fluency* or *ease of processing* hypothesis of aesthetic liking [18,34–37]. Elegance defies both physical gravity and intellectual difficulty: despite their enormous physical weight, elegant bridges spanning over miles can appear light, and elegant solutions of very difficult intellectual problems similarly come with an air of simplicity and lightness. Importantly, it is not just cognitive fluency, but, more specifically, cognitive fluency combined with (a) pronounced impressions of perceptual lightness, (b) blends of refinement and highly artful simplicity, and (c) a strong factor of exquisiteness/preciousness that renders elegance a highly distinct variant of fluency-supported aesthetic appeal.

#### (2) Elegance in personal appearance

Entries focus particularly on fashion/clothing (comprised of the listings for *fashion*, *dress*, *evening gown*, *clothing*, and *suit*), physical movement and posture (*dance*, *movement*, *gait*, *posture*), and personal looks (*svelte*, *looks*, *chic*). Even though men's suits are also listed, the data show a far stronger association of elegance with female (the entries being *woman*, *lady*, and *femininity*) than with male looks.

#### (3) Social selectivity and high socio-economic standing associated with elegance

Relevant entries include the words *distinguished*, *expensive*, *wealth*, und *glamour* (see S2 Fig, especially the upper part).

#### (4) Elegance in the arts

Even though art-related associations were listed far less frequently than those for personal elegance, the concept of elegance was associated with artistic achievements, too. However, only *dance*, *art*, *ballet*, *music*, and *poetry* (in descending order) passed the 5% cut-off we adopted for our analysis of the data.

#### Physical build and gait of elegant individuals

Prototypical physical features regarding elegant women are, in descending order, *slender*, *delicate*, *thin*, and at least *mean height*; for elegant men, *athletic*, *slender*, and *tall* are the most frequently expected features. Only between 0.0% and 6.7% percent of the participants associated an elegant physical build also (but by no means exclusively) with the experimenter-selected attributes *corpulent*, *portly*, *plump* and *stocky*. Ratings for *delicate* and *athletic* show the most pronounced dissociation between female and male elegance (see S4 Fig). The data for an elegant physical build of men are associated with tall body height and medium leg length, whereas those for an elegant physical build of women show, inversely, a stronger association with medium body height and long legs (see S5 Fig). Hence slenderness and long legs, while prototypical expectations for an elegant physical build in both women and men, appear to play an even more pronounced role for female than for male elegance.

Regarding the elegance of gait (for which we did not collect separate data sets for women and men), the characteristics *upright carriage*, *fluent*, and *light* were the most important, followed by *dancerly* and *elastic* (for all details see S1 Table).

#### Non-physical person variables associated with an elegant appearance

The elegance-dependent attributions we obtained for non-physical person variables include cognitive and verbal capacities (*intelligent*, *eloquent*, *creative*, *witty*), acquired cultural capital (*cultured*, *educated*, *self-confident*; for the notion of “cultural capital”, see [38]), prosocial and moral characteristics (*correct*, *sociable*, *conscientious*, *responsible*, *honest*, *caring*), social habitus (*polite*, *charming*, *gallant*, *open*) and social/professional success (*successful*, *rich*) (for all details see S2 Table).

These elegance-driven inferences can be considered an analogue to the *physical attractiveness stereotype*, i.e., to the attribution of several personality traits dependent on physical attractiveness [30–32]. Of course, such attributions are likely to be misattributions in many cases, as is true of all stereotypes. However, the goal of the present study was precisely to identify prototypical elegance-driven attributions not only of physical, but also of non-physical person variables. (A systematic comparison of the associations found for physical attractiveness and elegance was beyond the goals of our study.)

#### Age-dependency of sexy, beautiful, and elegant personal looks

**Fig 1:**
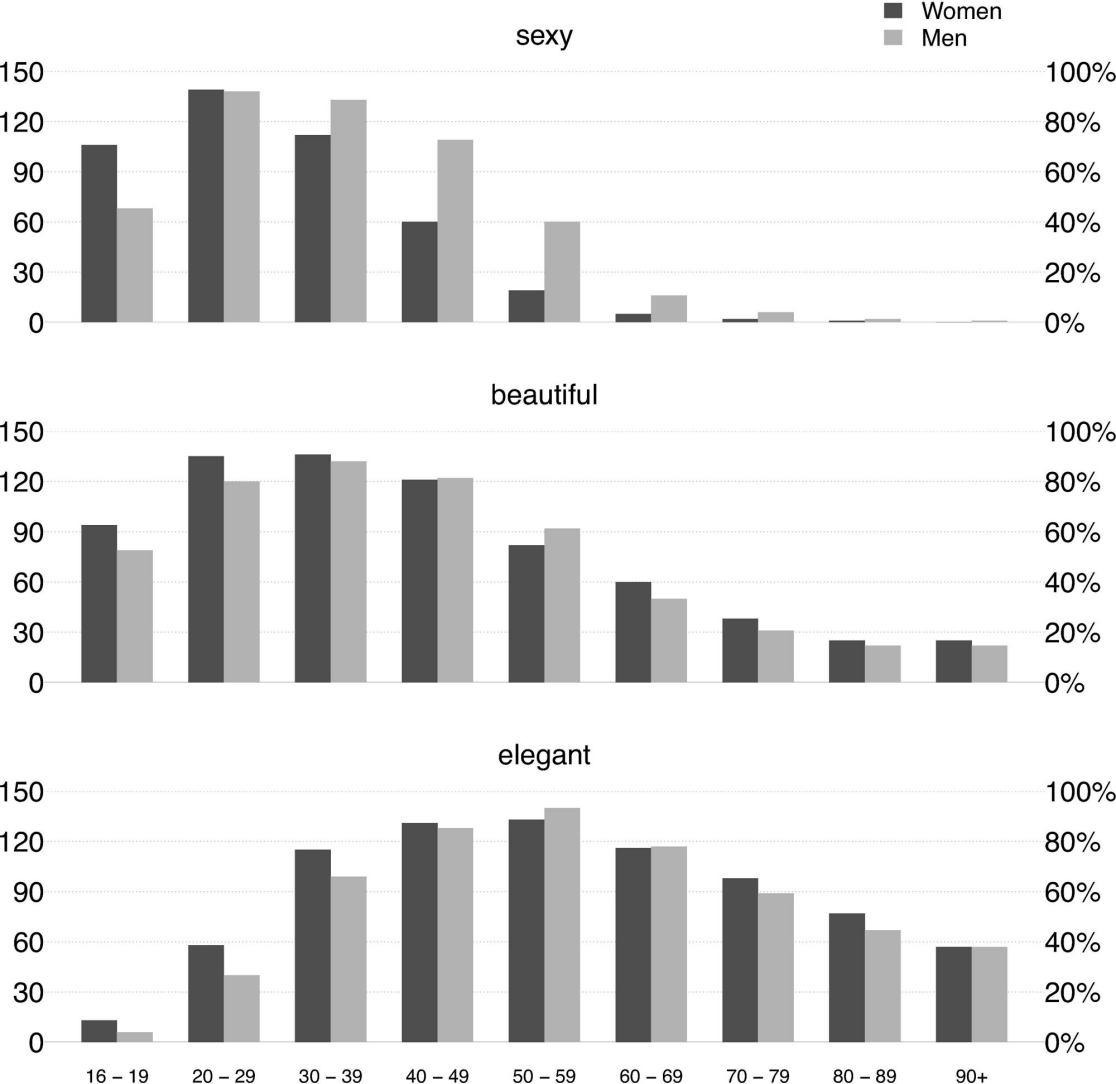
Subjectively perceived age-dependency of sexy, beautiful, and elegant personal looks. Bars represent the number of participants indicating that in the respective age ranges women (dark grey) and men (light grey) are likely to be perceived as elegant (lower part), beautiful (middle part), and sexy (upper part). Multiple entries for age range per concept and gender were possible.

The next target variable of our study was the factor of age. As explained in the Method section, the task was expected to yield information about *relative differences* in our participants’ spontaneous *attributions* of beauty, elegance, and sexiness (or absence thereof) to personal looks dependent on age, but not about absolute frequencies of such looks. Fig 1 shows the responses per age range and attribute (beauty, elegant, sexy) for men and women, respectively.

Logistic mixed-effects regression models predicted probabilities of responses in a full factorial design while accounting for non-independence of observations within participants due to multiple responses (the 90+ age category was omitted due to an empty cell). Probabilities of responses differed between attributes, age ranges, and gender (three-way interaction, χ²(14) = 39.4, *p* = .0003). Sidak-corrected post-hoc comparisons showed that significant gender differences were observed for sexiness attributions at some age ranges, with higher female sexiness at age 16–19 (*p* < .0001) and higher male sexiness at age 30–39 (*p* = .010), 40–49 (*p* < .0001), and 50–59 (*p* < .0001), but not for elegance (*p*s > .23) and beauty attributions (*p*s > .15) at any age.

At the same time, the data for sexiness and beauty attributions dependent on age-ranges are descriptively far more similar to one another than they are to those for elegance attributions. The latter reach peak levels only far later in age, with the ages 50 to 59 representing the very peak (contrast to sexiness: *p* < .0001; to beauty: *p* < .0001), and fairly high chances of being considered elegant extending even into old age. In contrast, elegance attributed to youths of 16–19 years is negligible (contrast to sexiness: *p* < .0001; to beauty: *p* < .0001), and remains significantly lower compared to attributions of beauty and sexiness (all *p*s ≤ .005) for the subsequent two decades. For the age range of 40–49, attributions of beauty and elegance show no significant difference anymore (*p* = .51), and sexiness attributions are significantly lower than those for elegance (*p* < .0001). Across all remaining age ranges, elegance attributions are significantly higher than those for sexiness and beauty (*p*s < .0001).

Thus, our data show a nearly perfect dissociation of elegant and sexy looks at both young and older ages. For beauty and elegance, the pattern of dissociation was structurally similar, but far less pronounced. Chances of being considered elegant at age 60 and older are roughly two times as high as chances of being considered beautiful and four times as high as chances of being considered sexy. Hence, in our data, sexiness and elegance of personal appearance largely diverge, with beauty representing a middle ground that show roughly the same amount of overlap with elegant and with sexy looks, respectively.

Whereas the parameters of elegant bodily looks (slenderness, long legs, at least mean height) largely converge with current (Western) ideals of physical attractiveness (beauty), elegance in personal appearance strongly deviates from the current idealization of beautiful youthful looks in one important respect: it is about culturally refined good looks not only of mature adults, but also of individuals in their advanced ages and even in very old age.

Overall, results show a pronounced nature-culture difference across the three categories. The “sexy”-attributions for women reach earlier high levels than those for men, are equal for both sexes at the age 20–29, and then decline for both sexes, albeit far more rapidly and drastically so for women than for men. This distribution is likely to reflect differences in the age-ranges in which women and men, respectively, are biologically most likely to have off-spring and can hence be considered sexually “attractive” in a narrower meaning.

It is all the more remarkable, then, how strongly beauty attributions depart from these biological constraints. The decline in beauty attributions is far less steep and swift than in sexiness attributions. Perhaps even more importantly, this decline does *not* show any larger differences for women and men across all age ranges. This supports the assumption that beauty attributions to persons are clearly and markedly different from attributions of sexual attractiveness, even though they are likely to also entail aspects of natural physical beauty.

Regarding elegance, the difference to sexiness attributions is even more pronounced. The data for the likelihood of elegance attributions show even far higher absolute levels than those for beauty until very high age, and significant gender differences are not found in our data from age 30 onward. In this sense, elegance appears to the most cultural and the most “age-fair” type of beauty.

Thus, as expected, sexiness and elegance attributions show the greatest dissociation, with beauty attributions covering the middle ground.

#### Elegance of verbal articulation and argumentation

The ratings obtained for elegant diction are high (in descending order) for *well-spoken*, *poised*, *meticulously articulated*, *nuanced wording*, *rich in vocabulary*, *exquisite vocabulary*, *eloquent*, *precise in expression*, *sophisticated*, *correct*, *pointed*, *quick-witted*, *rich in imagery*, and *playful*, and low for *complicated*, *verbose*, *stilted*, *long-winded*, and *monotonous* (for all details see S3 Table). Several of the high-ranking items of our questionnaire specifically apply to contexts of conversation and oral performance, particularly *quick-witted*, *meticulously articulated*, and partly also *poised*. Overall, the results indicate a nuanced persistence of the older notion of rhetorical elegance among contemporary individuals.

All data obtained in Study 1 were furthermore separately analyzed for differences dependent on the participants’ age and gender. Overall, the understanding of elegance was found to be very consistent and only marginally affected by these variables (for all details, see S4 Text).

## Study 2

Whereas the comparison of beauty, elegance and sexiness in Study 1 was limited to dimensions of personal appearance, Study 2 proceeded to a full-blown comparison of beauty, elegance, grace, and sexiness regarding which perceptual, affective, cognitive, and also social dimensions/associations are in general held to be distinctive of the four concepts. The comparison was performed using semantic differentials [27]. Participants were asked to characterize their understanding of beauty, elegance, grace(fulness), and sexiness, respectively, through ratings on 42 items presented on 7-point scales. The opposite ends of the 42 scales were antithetical adjectives.

### Methods

#### Participants

Four hundred forty-six students with a mean age of 23.1 years (*SD* = 7.32, median = 21, min = 16, max = 75) participated in Study 2 (262 female, 182 male, 2 undisclosed). Three hundred seventy-one were native speakers of German; 67 were bilingual, 7 non-native speakers, and 1 undisclosed. Each participant gave ratings for one of the target concepts only. We excluded the data (a) of the 1 undisclosed and the 7 non-native-speaking participants and (b) of 8 participants who completed less than 50% of the ratings. Thus, we ended up with 430 valid datasets (108 for beauty, 111 for elegance, 102 for grace, and 109 for sexiness).

#### Ethics statement

Study 2 was conducted in full accordance with the World Medical Association’s Declaration of Helsinki and the Ethical Guidelines of the German Association of Psychologists (DGPs). Participation was based on implicit rather than explicit informed consent; non-consenting individuals did not produce any data or did not return a questionnaire. We did not record this type of consent, in accordance with the rules for dispensing with recording/documenting informed consent that are stipulated in § C.III.6 of the Ethical Guidelines of the German Association of Psychologists [39]. (Note that for the completely anonymous data acquisition we performed, it is not only not mandatory, but also unusual at German universities to ask for written informed consent.)

#### Questionnaire

**Fig 2:**
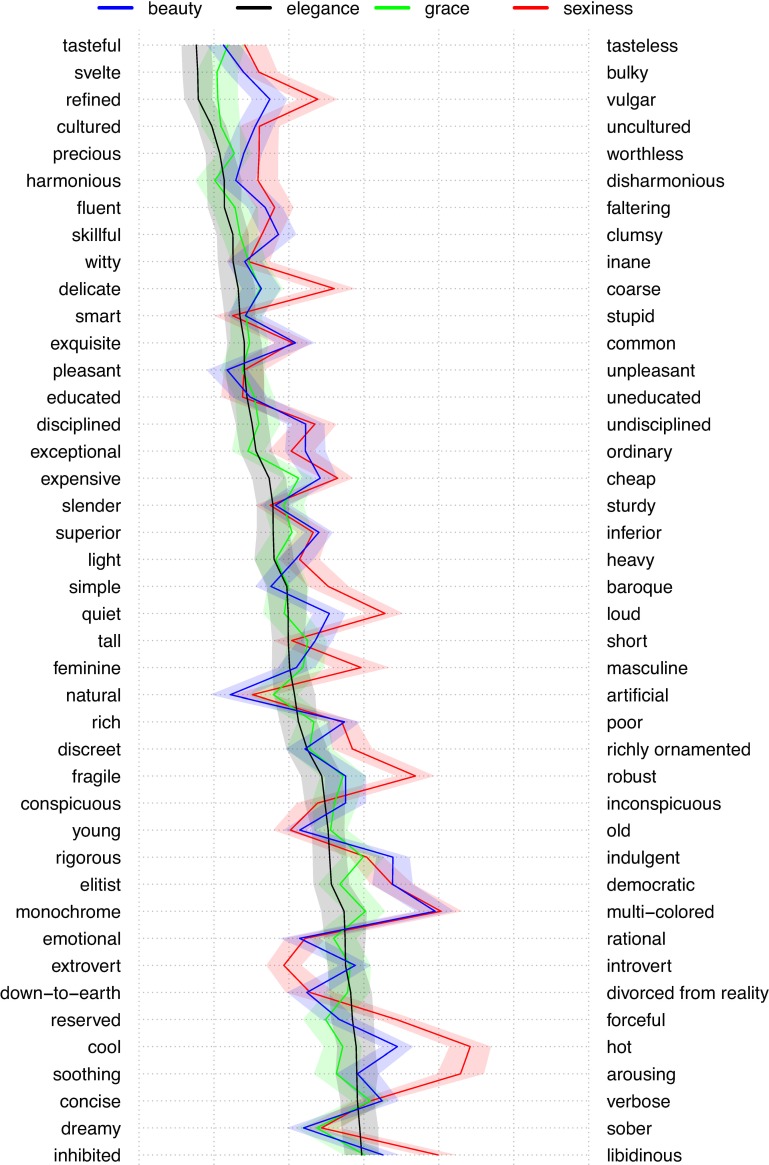
Semantic differential profiles for beauty, elegance, grace, and sexiness. Mean values plus 95% CI band for the concepts beauty (blue), elegance (black), gracefulness (green), and sexiness (red).

Based on intuitive and theory-based anticipations, co-occurrences in linguistic corpora, and the free associations of Study 1, we first collected 145 pairs of adjectives as candidate terms. The authors and colleagues (n = 6) rated these pairs with regard to the degree to which they might reveal differences between the concepts of beauty, elegance, grace, and sexiness. By selecting only those pairs that had a high mean and a low standard deviation in the ratings, we obtained 39 of the 42 adjective pairs listed in Fig 2. Three additional pairs were selected for theoretical reasons: “pleasant–unpleasant” as a classical marker of valence, “natural–artificial” because the nature-art distinction is of major importance in aesthetics in general, and “monochrome–multi-colored” because we wanted to check whether the very reduced color spectrum associated with elegance in the free association task in Study 1––only the entry “black” passed the 5% cut-off––could be replicated using a different method. (See at the Open Science Framework, https://osf.io/uqga9/, for the German and an English version of the questionnaire.)

#### Procedure

The data were collected at the end of several lecture classes (computer science, literary studies, biology, physics, sports, psychology, cultural anthropology) at Goethe University Frankfurt, Germany. Students were asked if they would volunteer to participate in the study, which only took a few minutes. They were explicitly informed about the task they were expected to perform (i.e., to rate, within two minutes, the concept of either beauty, grace, elegance, or sexiness on various scales, and to also indicate their gender, age, field of study, and native language), the anonymity of the data, the fully voluntary nature of their participation, and their right to withdraw from the study at any time. We handed out the questionnaire consisting of one sheet of paper only to those who volunteered and retrieved it after two minutes. No identifying personal information was obtained.

### Results and discussion

We report analyses of the overlap and differences between all four categories on three levels of resolution. First, we looked at overall differences by calculating the mean absolute differences based on all ratings obtained for all semantic differentials as well as calculating MANOVAs for each pair of concepts. Second, we performed an exploratory factor analysis and calculated for each pair of concepts separate MANOVAs for the items making up the seven individual factors. Finally, we identified the respective individual differentials on which each target concept converges with, and diverges from, the other target concepts.

#### Mean absolute differences between the target categories across all semantic differentials

The overall mean of the absolute differences across all 42 semantic differentials measured on 7-point scales are 0.442 (SD = 0.288) for beauty and elegance, 0.328 (SD = 0.243) for beauty and grace, 0.361 (SD = 0.349) for beauty and sexiness, 0.180 (SD = 0.130) for elegance and grace, 0.649 (SD = 0.417) for elegance and sexiness, and 0.562 (SD = 0.425) for grace and sexiness. Highlighting the role of beauty as a middle ground, the overall mean of the absolute differences between beauty and the other three categories are all within a narrow margin (ranging from 0.328 to 0.442). Comparing elegance and grace with sexiness yielded the highest mean differences (0.649 and 0.562, respectively), whereas elegance and grace show a particularly low mean of the absolute differences of only 0.180, and hence are barely distinguishable on our 42-item measure.

MANOVAs performed for pairwise comparisons of all target categories across all semantic differentials yielded a convergent picture. The highest significant difference was obtained for elegance and sexiness (Wilk’s λ = 0.310, Pillai’s trace = 0.690, *F*(42,109) = 5.78, *p <* .001), followed by the difference of grace and sexiness (Wilk’s λ = 0.316, Pillai’s trace = 0.684, *F*(42,105) = 5.41, *p <* .001). The lowest difference was again found for elegance and grace (Wilk’s λ = 0.714, Pillai’s trace = 0.286, *F*(42,109) = 1.04, *p* = .42) and did not reach significance. The other three comparisons yielded levels of significant differences that cover the middle ground between these extremes: beauty and elegance (Wilk’s λ = 0.460, Pillai’s trace = 0.540, *F*(42,110) = 3.08, *p <* .001), beauty and sexiness (Wilk’s λ = 0.498, Pillai’s trace = 0.502, *F*(42,106) = 2.55, *p* < .001), and beauty and grace (Wilk’s λ = 0.533, Pillai’s trace = 0.467, *F*(42,106) = 2.21, *p* < .001).

#### Factor analysis across all four data sets

We performed a post-hoc exploratory factor analysis across all four data sets (beauty, elegance, grace, and sexiness) using the R psych package [40]. The overall KMO index was .85, indicating suitability for factor analysis [41]. Parallel analysis [42,43] yielded seven factors to be extracted. We extracted these seven factors using principal axis method with oblique rotation. The factors can be interpreted as (1) Tastefulness/Fluency, (2) Affective Habitus/Arousal, (3) Cultural Sophistication, (4) Intellectual Rigor, (5) Lightness/Slenderness, (6) Exquisiteness, and (7) Delicacy/Femininity (see S6 Table, for loadings of the 42 items on the seven factors).

In accordance with the findings of the analyses of the mean absolute differences, MANOVAs (Bonferroni-corrected for multiple testing; see Table 2 for a summary and S7 Table for statistical details) revealed that elegance differs significantly from grace on none of the seven factors, from sexiness on all factors, and from beauty on an intermediate number (four) of factors (Tastefulness/Fluency, Intellectual Rigor, Lightness/Slenderness, and Exquisiteness). In contrast, beauty differs from sexiness only on the two factors Affective Habitus/Arousal and Delicacy/Femininity. Grace and sexiness are significantly different on all factors except factor 3 (Cultural Sophistication).

**Table 2:** Summary results for the pairwise comparison of the four concepts for each of the seven factors.

	Elegance vs Beauty	Grace vs Beauty	Beauty vs Sexiness	Elegance vs Grace	Elegance vs Sexiness	Grace vs Sexiness
(1) Tastefulness/Fluency	**+**	**+**	**·**	**·**	**+**	**+**
(2) Affective Habitus/Arousal	**·**	**−**	**−**	**·**	**−**	**−**
(3) Cultural Sophistication	**·**	**·**	**·**	**·**	**+**	**·**
(4) Intellectual Rigor	**+**	**+**	**·**	**·**	**+**	**+**
(5) Lightness/Slenderness	**+**	**+**	**·**	**·**	**+**	**+**
(6) Exquisiteness	**+**	**+**	**·**	**·**	**+**	**+**
(7) Delicacy/Femininity	**·**	**·**	**+**	**·**	**+**	**+**

Note. The sign ‘**·**’ indicates absence of significant differences (Bonferroni-corrected for multiple testing) between the two concepts compared in the six vertical columns. Significantly higher mean values for the concepts represented in the uppermost line of the table (compared to those listed below them) are indicated by ‘+’, and significantly lower values by ‘−’.

#### Differences between the four target categories dependent on individual semantic differentials

Finally, we compared all four categories (beauty, elegance, grace, and sexiness) pairwise for differences and convergences on the level of all 42 semantic differentials. We did so by computing Bayes factors (as alternative to the conventional t-test) [44–46] for each adjective pair using the ttestBF function of the R package BayesFactor [47]. The default settings allowed conclusions regarding evidence for significant differences, absence thereof (no difference), and inconclusive results (for all details, see S5 Text). We here highlight the most important findings regarding positive evidence for differences and absence of differences.

Looking at individual items, beauty as compared to elegance is conceived as being more *multi-colored*, *natural*, and *dreamy*, and less *refined*, *rigorous*, *elitist*, *disciplined*, *exquisite*, *expensive*, *exceptional*, *rich*, *svelte*, and *skillful*. Beauty as compared to sexiness scores higher on the items *soothing*, *delicate*, *fragile*, *feminine*, *simple*, *reserved*, *quiet*, *refined* and *discreet*, and lower on the items *libidinous*, *extroverted* and *hot*. Beauty as compared to grace is conceived as more *multi-colored*, *natural*, *down to earth*, *younger*, *and hotter*, and as less *elitist*, *rich*, *skillful*, *quiet*, *exquisite*, *disciplined*, *refined* and *exceptional*. Given the close affinity of elegance and grace, it is not surprising––and highlights the consistent quality of our data––that the differences obtained for beauty and grace largely overlap with those for beauty and elegance.

Elegance compared to sexiness is conceived as less *arousing*, less *extroverted*, less *libidinous*, less *hot*, less *loud*, less *multi-colored*, less *natural*, less *robust*, and more *tasteful*, more *fluent*, more *fragile*, *richer*, *older*, *svelter*, more *elitist*, more *refined*, more *disciplined*, more *cultured*, more *exquisite*, more *rigorous*, more *expensive*, more *feminine* and more *delicate* (see Fig 2). Similarly, grace compared to sexiness is conceived as less *arousing*, less *extroverted*, less *libidinous*, less *hot*, less *loud*, less *multi-colored*, less *robust*, and more *fluent*, more *fragile*, *richer*, *older*, *svelter*, more *elitist*, more *refined*, more *disciplined*, more *cultured*, more *exquisite*, more *expensive*, more *feminine* and more *delicate*.

Notably, all items that distinguish beauty and sexiness likewise distinguish elegance and sexiness; however, the degree of the differences is far greater between elegance and sexiness than between beauty and sexiness. Moreover, there are roughly three times as many semantic differentials showing differences between elegance and sexiness compared to those showing differences for beauty and sexiness. Regarding the semantic differential *multi-colored* vs. *monochrome*, ratings for elegance lean far more strongly to the “monochrome” end than those for “beauty” and “sexiness;” at the same time, absolute levels are near the midpoint of the scale, suggesting that elegant objects can well include objects more colorful than the black dress.

Regarding elegance and grace, positive evidence for differences was obtained only for four pairs: grace appears to be conceived as slightly less *sober*, *rigorous*, *tasteful* and *expensive* than elegance. This corroborates the very close affinity of elegance and grace obtained in the free association task of Study 1. At the same time, the differences we found are well in line with the concept of grace in classical aesthetics. After all, grace––in contrast to elegance––is also attributed to movements of children and hence to phenomena which do not necessarily involve intellectual rigor and “high” taste, let alone high purchasing power. (Accordingly, we had expected grace to differ from elegance on the semantic differential of *natural vs*. *artificial*. However, though the descriptive difference was showing the expected pattern of a higher naturalness of grace, Bayes Factors yielded evidence that elegance and grace do not differ in this regard.)

In sum, favoring Lord Kames’ definition of grace as “elegance in motion” over Schiller’s definition as “beauty in motion,” our results show a stronger affinity of grace with elegance than with beauty. At the same time, as both elegance and grace are closely related with beauty, Schiller’s definition is justified as well.

Like in Study 1, we additionally performed separate analyses that tested all four concepts for gender differences. Results show no statistically significant difference; this holds both for each of the 42 semantic differentials when analyzed separately, and for their entirety (for details, see S6 Text). For sexiness, the MANOVA yielded a significant difference between men and women, mostly driven by the items *feminine-masculine*, with men rating sexiness as being more *feminine* and women as more *masculine*, and for *tall-short*, with women associating sexiness with greater body height (*tall*) than men.

## General discussion

Our study shows that elegance and grace differ very strongly and categorially from sexiness, yet that all these categories still show much overlap with the broader category of beauty. Each of these four categories allows for a precise comparative definition regarding characteristic dimensions of aesthetic appeal and is, moreover, associated with distinct cognitive, affective and partly also social implications.

From a methodological point of view, we would like to note that linguistic corpus analyses alone could barely have tapped into the distinct cognitive and affective profiles of the four concepts under scrutiny in nearly as controlled, directly comparable and multi-dimensional a fashion as their projection onto 42 carefully chosen bipolar dimensions which all allow for subtle graded distinctions on scales ranging from 1 to 7.

Elegance appears to be a particularly fruitful topic for future research, as it is a very distinct aesthetic virtue that is still found across multiple domains of both visual and verbal aesthetics and is lexicalized across many languages and cultures. Reflecting its importance, the concept of elegance is, in fact, frequently used in journals on fashion, design, and architecture. However, previous scientific efforts to explicate the meaning of elegance are largely confined to elegance in scientific proofs and software programming [48–54]. In contrast, visual and verbal elegance has to date gone almost completely untreated both in philosophical and in empirical aesthetics. Accordingly, neither the Oxford *Encyclopedia of Aesthetics* [55] nor a German six-volume dictionary of key concepts of aesthetics [56] include an entry for “elegance.”

The preeminent importance of dimensions of fluency, lightness, delicacy (slenderness), and simplicity for the mental construct of elegance strongly support the notion that the stipulations of the *processing fluency* or *ease of processing* hypothesis of aesthetic liking [18] entail great potential for future research specifically on elegance. In a similar vein, grace remains a relevant aesthetic virtue regarding dance and other movements.

In sum, our data strongly suggest that it is time to put hitherto neglected concepts closely related to beauty––specifically, elegance and grace––on the agenda of research in aesthetics. Considering these nuances would help to prevent beauty ratings from being used as too broad and hence too vague a standard currency in empirical aesthetics. Importantly, our study provides concrete guidance as to which rating items could be used to collect multi-item measures of elegance, grace, beauty, and sexiness.

### Limitations and future directions

The results obtained in our two studies exclusively reveal the current understanding of the four target terms in a Western cultural context. Specifically, the strong association with intellectual rigor and impressions of simplicity we found in our data for elegance is likely to be influenced by modernist reinterpretations of elegance as in Bauhaus architecture and design and the concomitant distancing from an aesthetics of ornament [57]. Elegant aristocratic fashions were often highly ornamental and playful, and far less purist and austere. In cultures in which modernist design and architecture had no comparable roles, the current understanding of elegance may well be different. On a similar note, the degree to which the concepts of grace and sexiness vary across languages and cultures also warrants further investigation.

Our questionnaire did not specifically target any negative attributions of personality traits that might also be associated with an elegance personal appearance. However, just like physical attractiveness research has revealed not only positive, but also some negative trait attributions dependent on perceived attractiveness ([58–61], pp. 241–252), similar adverse effects on person perception may well be obtained for elegance, too, if targeted by appropriate scales.

The null results regarding gender and age differences (as reported in S4 and S6 Texts in the Supporting Information) cannot readily be generalized, as our samples were not balanced concerning these variables. Moreover, in our sample, participants aged 30 and higher make up only 25% of the sample and are hence relatively underrepresented.

Our two studies—though guided by fairly pronounced expectations—are to a large extent exploratory in nature. Testing specific hypotheses derived from our present findings would be a next step. Statistical analyses of elegant objects and features of movement are further very interesting topics for future research.

Considering that elegance is strongly associated with beauty, but that by no means all beauty is elegant, the question arises whether neural correlates of perceiving elegance can partly be dissociated from those that have been reported for perceived beauty [62–67].

Last, but not least, our two studies exclusively elucidate the *mental constructs* which individuals have acquired regarding beauty, elegance, grace, and sexiness. In order to consolidate these findings, the distinct pattern of overlaps and differences between the four categories would need to be replicated by studies in which participants rate preselected sets of beautiful, elegant, graceful and sexy stimuli on a broader range of identical scales.

## Conclusion

The present study elucidates affinities and differences between beauty, elegance, grace, and sexiness on a rich set of cognitive, affective, age- and gender-related, and social implications. These comparative distinctions have great potential to help overcome the relative vagueness with which a barely defined concept of beauty is largely dominating empirical studies in aesthetics.

## Supporting information

S1 Text Collocates of the term “elegant”. (DOCX)

S2 Text Details of the participant sample of Study 1. (DOCX)

S3 Text Exploratory analyses of the free association data. (DOCX)

S4 Text Testing for effects of gender and age. (DOCX)

S5 Text Bayes factor analyses for pair wise compassion of the four categories. (DOCX)

S6 Text Analysis of the semantic differential ratings for beauty, elegance, grace, and sexiness by gender. (DOCX)

S1 Fig Dendrograms of hierarchical cluster analyses. (PNG)

S2 Fig Classical multi-dimensional scaling. (PNG)

S3 Fig Visualizing the co-occurrences as a network. (PNG)

S4 Fig Aspects of physical build. (PDF)

S5 Fig Expected height and leg length. (PDF)

S1 Table Descriptive statistics for gait. (XLSX)

S2 Table Descriptive statistics for person variables. (XLSX)

S3 Table Descriptive statistics for elegant diction. (XLSX)

S4 Table Descriptive statistics for elegant objects. (XLSX)

S5 Table Most frequently mentioned elegant persons (celebrities). (XLSX)

S6 Table Exploratory factor analysis. (XLSX)

S7 Table Results of pairwise comparisons (MANOVAs) for the seven factors. (XLSX)
