# Supplementary material for: Beauty, elegance, grace, and sexiness compared
Source: PLoS One. 2019 Jun 21;14(6):e0218728. doi: 10.1371/journal.pone.0218728 (PMC6588248; doi:10.1371/journal.pone.0218728)
Supplement: S1 Text — (DOCX) [file pone.0218728.s001.docx]

### S1 Text. Collocates of the term “elegant”

In order to retrieve lists of elegant objects, we ran two queries on online corpora, one on the *Corpus of Contemporary American English* (COCA; <https://corpus.byu.edu/coca/>) and the other on the *Wortprofil* of the *Digitales Wörterbuch der Deutschen Sprache* (DWDS; <https://www.dwds.de/wp>; [1]). The results of both queries are reported in table below.

For the COCA-query, we searched for collocates of “elegant” with the restrictions that the collocates should be nouns (PoS NOUN) and occur as the first word after the search term.


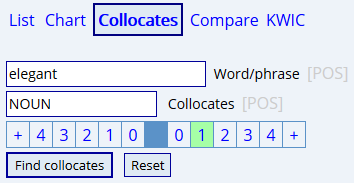


For the DWDS-query, we set the number of returned entries up to 100. Potentially elegant objects are designated by words which take “elegant” as an adjective attribute.

### Table. Collocates for “elegant”

|  | COCA |  |  | DWDS |  |  |
| --- | --- | --- | --- | --- | --- | --- |
| **#** | **collocates** | **freq.** |  | **adj. attr. of** | **logDice** | **freq.** |
| 1 | solution | 86 |  | Anzug | 8.2 | 355 |
| 2 | way | 73 |  | Schwung | 7.9 | 210 |
| 3 | man | 63 |  | Dame | 7.7 | 470 |
| 4 | woman | 62 |  | Erscheinung | 7.6 | 262 |
| 5 | dining | 48 |  | Kleid | 7.5 | 209 |
| 6 | dinner | 38 |  | Kostüm | 7.5 | 161 |
| 7 | restaurant | 36 |  | Lösung | 7.2 | 651 |
| 8 | home | 34 |  | Kleidung | 7.0 | 119 |
| 9 | simplicity | 32 |  | Herr | 7.0 | 329 |
| 10 | hotel | 31 |  | Restaurant | 6.9 | 148 |
| 11 | homes | 29 |  | Abendkleid | 6.8 | 77 |
| 12 | design | 27 |  | Hosenanzug | 6.8 | 75 |
| 13 | fingers | 26 |  | Design | 6.8 | 105 |
| 14 | lady | 25 |  | Hotel | 6.8 | 183 |
| 15 | lines | 24 |  | Bogen | 6.7 | 99 |
| 16 | look | 24 |  | Villen | 6.7 | 100 |
| 17 | style | 23 |  | Schuhe | 6.5 | 90 |
| 18 | house | 21 |  | Salons | 6.5 | 72 |
| 19 | room | 21 |  | Ausweg | 6.5 | 76 |
| 20 | solutions | 20 |  | Ambiente | 6.4 | 75 |
| 21 | hand | 19 |  | Stil | 6.3 | 167 |
| 22 | rooms | 19 |  | Weise | 6.3 | 414 |
| 23 | clothes | 18 |  | Mantel | 6.3 | 70 |
| 24 | manner | 18 |  | Roben | 6.2 | 51 |
| 25 | surroundings | 18 |  | Garderobe | 6.2 | 48 |
| 26 | universe | 18 |  | Outfit | 6.1 | 52 |
| 27 | presentation | 17 |  | Kurve | 6.1 | 62 |
| 28 | food | 15 |  | Mode | 6.1 | 60 |
| 29 | restaurants | 15 |  | Boutiquen | 6.1 | 45 |
| 30 | setting | 15 |  | Formulierung | 6.0 | 72 |
| 31 | women | 15 |  | Zweireiher | 6.0 | 40 |
| 32 | dessert | 14 |  | Hut | 5.9 | 55 |
| 33 | prose | 14 |  | Stilist | 5.9 | 40 |
| 34 | silk | 14 |  | Art | 5.9 | 252 |
| 35 | apartment | 13 |  | Limousine | 5.8 | 47 |
| 36 | figure | 13 |  | Leichtigkeit | 5.8 | 44 |
| 37 | meal | 13 |  | Bar | 5.8 | 43 |
| 38 | attire | 12 |  | Linienführung | 5.8 | 36 |
| 39 | bow | 12 |  | Abgang | 5.7 | 43 |
| 40 | designs | 12 |  | Abendroben | 5.6 | 32 |
| 41 | dress | 12 |  | Bewegungen | 5.6 | 137 |
| 42 | mansion | 12 |  | Methode | 5.6 | 106 |
| 43 | office | 12 |  | Frau | 5.5 | 471 |
| 44 | silver | 12 |  | Cafés | 5.5 | 37 |
| 45 | evening | 11 |  | Karosserie | 5.5 | 32 |
| 46 | features | 11 |  | Prosa | 5.4 | 34 |
| 47 | forms | 11 |  | Yachten | 5.4 | 31 |
| 48 | men | 11 |  | Universum | 5.4 | 31 |
| 49 | place | 11 |  | Dreiteiler | 5.3 | 26 |
| 50 | town | 11 |  | Form | 5.3 | 231 |
| 51 | building | 10 |  | Büro | 5.3 | 47 |
| 52 | gentleman | 10 |  | Lokal | 5.3 | 32 |
| 53 | gown | 10 |  | Seebad | 5.3 | 26 |
| 54 | head | 10 |  | Französisch | 5.2 | 25 |
| 55 | living | 10 |  | Bau | 5.2 | 53 |
| 56 | neck | 10 |  | Techniker | 5.2 | 28 |
| 57 | recipes | 10 |  | Läden | 5.2 | 40 |
| 58 | salon | 10 |  | Sprung | 5.2 | 42 |
| 59 | variation | 10 |  | Fassade | 5.2 | 39 |
| 60 | experiments | 9 |  | Abendgarderobe | 5.1 | 22 |
| 61 | hotels | 9 |  | Brücke | 5.1 | 38 |
| 62 | ladies | 9 |  | Kleider | 5.1 | 24 |
| 63 | lobby | 9 |  | Maßanzug | 5.1 | 22 |
| 64 | parties | 9 |  | Auftreten | 5.1 | 34 |
| 65 | society | 9 |  | Boulevard | 5.1 | 23 |
| 66 | space | 9 |  | Drehung | 5.1 | 23 |
| 67 | touch | 9 |  | Lady | 5.0 | 24 |
| 68 | art | 8 |  | Interieur | 5.0 | 23 |
| 69 | alternative | 8 |  | Trick | 5.0 | 33 |
| 70 | buildings | 8 |  | Pose | 5.0 | 25 |
| 71 | cocktail | 8 |  | Geschäfte | 5.0 | 118 |
| 72 | country | 8 |  | Manier | 5.0 | 29 |
| 73 | facade | 8 |  | Heber | 5.0 | 21 |
| 74 | face | 8 |  | Pumps | 5.0 | 21 |
| 75 | form | 8 |  | Wohnung | 5.0 | 77 |
| 76 | furnishings | 8 |  | Wagen | 5.0 | 42 |
| 77 | garden | 8 |  | Silhouette | 5.0 | 22 |
| 78 | glass | 8 |  | Einkaufsstraße | 5.0 | 21 |
| 79 | manners | 8 |  | Schlichtheit | 5.0 | 21 |
| 80 | name | 8 |  | Flieger | 5.0 | 23 |
| 81 | people | 8 |  | Paar | 4.9 | 48 |
| 82 | structure | 8 |  | Geste | 4.9 | 46 |
| 83 | suit | 8 |  | Konstruktion | 4.9 | 31 |
| 84 | wife | 8 |  | Handbewegung | 4.9 | 21 |
| 85 | addition | 7 |  | Variante | 4.9 | 53 |
| 86 | beauty | 7 |  | Verbeugung | 4.9 | 20 |
| 87 | book | 7 |  | Architektur | 4.9 | 35 |
| 88 | brick | 7 |  | Weine | 4.9 | 35 |
| 89 | city | 7 |  | Unsinn | 4.9 | 22 |
| 90 | creatures | 7 |  | Experimenten | 4.8 | 36 |
| 91 | decor | 7 |  | Wendung | 4.8 | 29 |
| 92 | dish | 7 |  | Übergänge | 4.8 | 27 |
| 93 | drawing | 7 |  | Pariser | 4.8 | 21 |
| 94 | hands | 7 |  | Gentleman | 4.8 | 19 |
| 95 | line | 7 |  | Tänzer | 4.8 | 23 |
| 96 | model | 7 |  | Deutsch | 4.8 | 22 |
| 97 | outfit | 7 |  | Toilette | 4.8 | 21 |
| 98 | piece | 7 |  | Umschreibung | 4.8 | 19 |
| 99 | salons | 7 |  | Landhaus | 4.8 | 19 |
| 100 | side | 7 |  | Kombination | 4.8 | 29 |

***References***

1. Didakowski J, Geyken A. From DWDS Corpora to a German Word Profile – Methodological Problems and Solutions. 2014. In: Vernetzungsstrategien, Zugriffsstrukturen und automatisch ermittelte Angaben in Internetwörterbüchern [Internet]. Mannheim: Institut für Deutsche SpracheOnline publizierte Arbeiten zur Linguistik; [39‒47].
