# Supplementary material for: Beauty, elegance, grace, and sexiness compared
Source: PLoS One. 2019 Jun 21;14(6):e0218728. doi: 10.1371/journal.pone.0218728 (PMC6588248; doi:10.1371/journal.pone.0218728)
Supplement: S2 Text — (DOCX) [file pone.0218728.s002.docx]

### S2 Text. Details of the participant sample of Study 1

Asked for their own attitude towards elegance, participants reported a medium self-relevance of being perceived as elegant (*M* = 3.88, *SD* = 1.67), a low to medium frequency of dressing themselves elegantly (*M* = 3.63, *SD* = 1.40, on a scale from 1 = not at all to 7 = very often), and a relatively high liking for elegant clothing (*M* = 5.09, *SD* = 1.67). Thus, aesthetic responsiveness to elegance was high, even though both the reported self-relevance and frequency of dressing elegantly were substantially lower than the aesthetic liking ratings.

We divided the sample by splitting up the participants into a group of people younger than 30 years and a second group 30 years and older (109 and 41, respectively; see the following histogram for the age distribution). We found no gender and age differences in the data regarding the above-reported personal attitudes towards elegance; however, the age group of 30-77 years reported higher levels of education and a significantly higher monthly income.
