# Supplementary material for: Beauty, elegance, grace, and sexiness compared
Source: PLoS One. 2019 Jun 21;14(6):e0218728. doi: 10.1371/journal.pone.0218728 (PMC6588248; doi:10.1371/journal.pone.0218728)

### S3 Text. Exploratory analyses of the free association data

We used the (preprocessed) word associations that were mentioned by at least 5% of the participants to create a matrix with 43 columns for the associations and 148 rows for the participants. (Two participants had not listed any of the 43 associations and were therefore not included in these analyses). In this matrix, the numbers 1 vs 0 indicate the presence vs. absence of a word in the lists of the participants. Based on this matrix of co-occurrences we computed a (dis-)similarity matrix using the Jaccard-coefficient [1] (For hierarchical cluster analyses and multidimensional scaling, we used the dissimilarity matrix, for the network analysis the similarity matrix.)

Hierarchical cluster analyses with Ward’s method or complete linkage-method showed upon visual inspection either two main clusters or 10 more fine-grained clusters (see S1 Fig). Using different methods to determine the numbers of clusters [2], yielded no conclusive results regarding the optimal number of clusters.

A (classical) multi-dimensional scaling of the dissimilarity matrix for two dimensions (see S2 Fig) showed a spatial arrangement (roughly) reflecting the features and aspects of elegance (depicted more on the left side of the MDS plot) and the domains and objects classes which prototypically allow for an attribution of elegance (depicted more on the right side of the MDS plot). The network visualization (S3 Fig) shows the multiple connections between the associations, reflecting the complex and fine-grained association structure.

***References***

1. Real R, Vargas JM, Olmstead R. The Probabilistic Basis of Jaccard's Index of Similarity. Systematic Biology. 1996;45(3):380‒5. doi: 10.1093/sysbio/45.3.380.

2. Charrad M, Ghazzali N, Boiteau V, Niknafs A. NbClust: An R Package for Determining the Relevant Number of Clusters in a Data Set. Journal of Statistical Software. 2014;61(6):1‒36.

### S1 Figure. Dendrograms of hierarchical cluster analyses


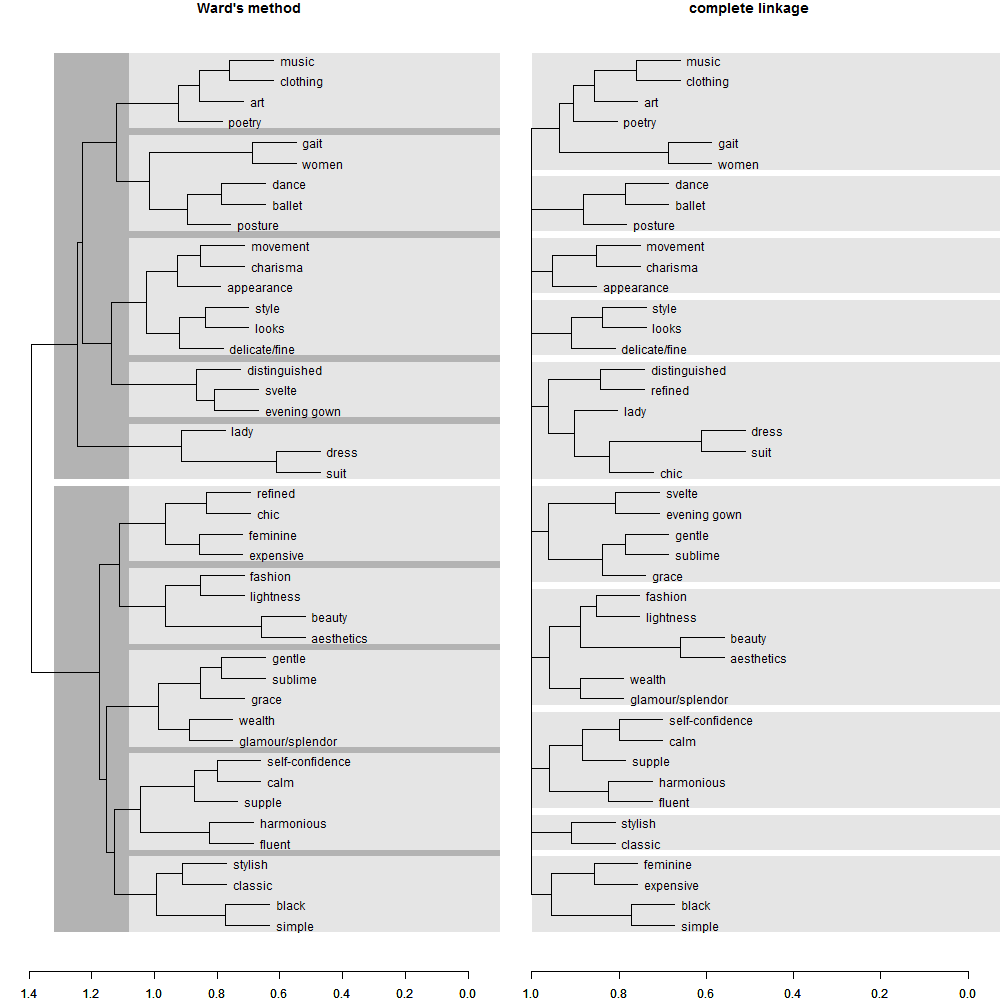


### S2 Figure. Classical multi-dimensional scaling


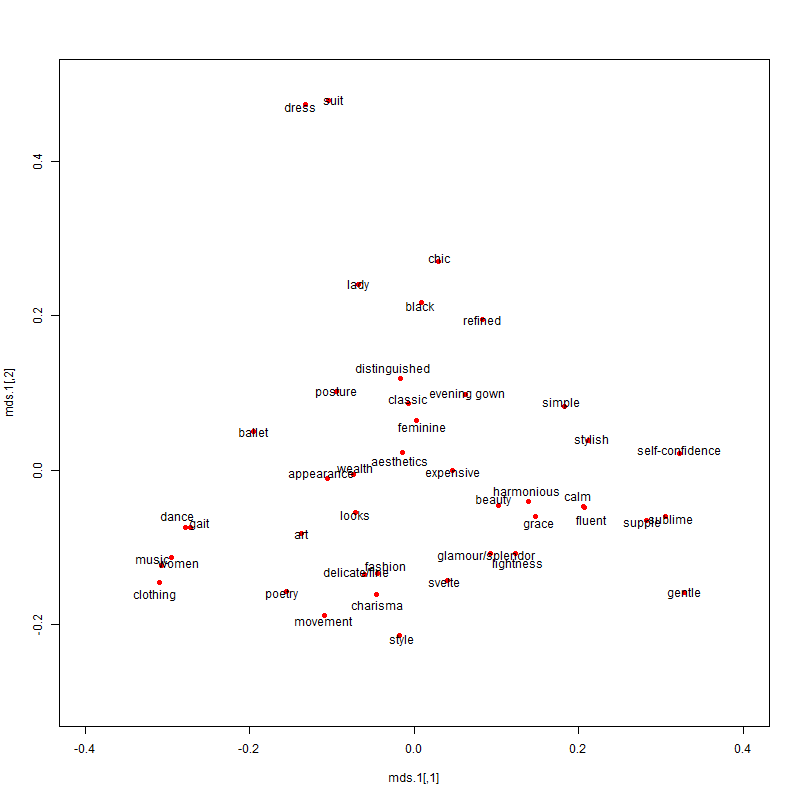


### S3 Figure. Visualizing the co-occurrences as a network

Network based on Jaccard-similarity scores. Coloring is based on hierarchical clustering with complete linkage (see S1 Figure, right panel). Line thickness represents Jaccard-similarity score between two associations (depicted only for scores greater 0.1). Circle size is proportional to the CSI-value (see Table 1 in the main text).
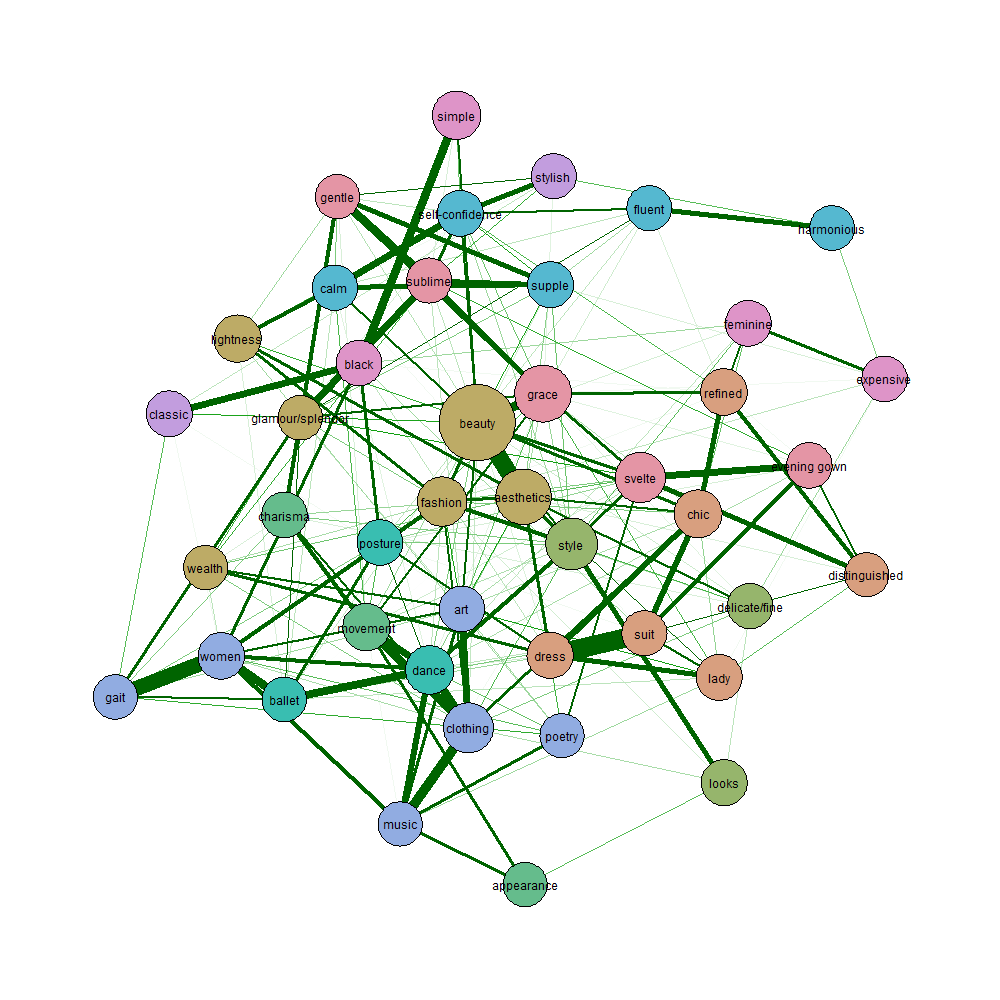

Supplement: S3 Text — (DOCX) [file pone.0218728.s003.docx]
