# Supplementary material for: Beauty, elegance, grace, and sexiness compared
Source: PLoS One. 2019 Jun 21;14(6):e0218728. doi: 10.1371/journal.pone.0218728 (PMC6588248; doi:10.1371/journal.pone.0218728)
Supplement: S4 Text — (DOCX) [file pone.0218728.s004.docx]

### S4 Text. Testing for effects of gender and age

#### **Gender differences**

Expectations regarding elegant physical build (χ^2^(20) = 9.11, *p* = .98, body height, and leg length (χ^2^(9) = 6.88, *p* = .65) did not differ between female and male participants. We also found no significant gender difference for any of the seven age ranges for which we collected expectations regarding the likelihood of elegance, beauty and sexiness in personal appearance (χ^2^(52) = 32.65, *p* = .98).

Regarding the ratings of personal attributes, there was overall no significant difference between men and women (Wilk’s λ = 0.824, *F*(23,125) = 1.16, *p* = .29). However, for one of the 23 items did we find a significant difference: men attributed the predicate *rich* to a lesser degree to elegant individuals than did women (t(150) = 3.3, *p* = .041, Bonferroni-corrected). Women had higher means for all items regarding gait (Wilk’s λ = 0.866, *F*(6,142) = 3.65, *p* < .01) with the exception of *hunched (gebückt)*. However, this difference only means that they expressed the same trend to a stronger degree. Ratings for elegant diction showed no significant difference (Wilk’s λ = 0.867, *F*(20,128) = 0.98, *p* = .49).

We only found significant gender differences that were in line with stereotypical gender expectations in the task that did not target aspects of the construct of elegance itself, but its application to individual object classes: Women rated hairstyles, hats, and underwear as more likely to be elegant and to be elegant to a higher degree, whereas men gave higher ratings to cars, bridges, and skyscrapers. Overall, these differences were significant both regarding how frequently men and women consider individual items of these object classes as elegant and to what degree they do so (Wilk’s λ = 0.793, *F*(14,134) = 2.49, *p* < .01 and Wilk’s λ = 0.722, *F*(14,134) = 3.68, *p* < .001).

In sum, the construct of elegance appears to be very convergent for men and women.

#### **Age differences**

We also separately analyzed the data of the 150 study participants by splitting them up into a group of participants younger than 30 years and a second group 30 years and older (109 and 41, respectively; see Text S1.1).

Overall, we found no significant differences between the age groups regarding the expectations for physical build (χ^2^(20) = 19.64, *p* = .48), body height and leg length (χ^2^(9) = 4.20, *p* = .90) and for the age-range-dependent attributions of elegance, beauty and sexiness (χ^2^(52) = 43.63, *p* = .79). Still, in the older group, age-related expectations of the likelihood of sexiness, beauty, and elegance in men and women shifted towards higher age. However, only the difference between younger and older participants regarding the age range of female sexiness (but not of male and female beauty and elegance, nor of male sexiness) reached statistical significance (χ^2^(7) = 25.9, *p* < .01):

Ratings regarding elegant gait (Wilk’s λ = 0.961, *F*(6,142) = 0.96, *p* = .45) did not differ significantly between younger and older participants.

The age groups differed in their ratings on personal attributes (Wilk’s λ = 0.694, *F*(23,125) = 2.42, *p <* .01). Specifically, the perceived elegance of personal appearance had a less powerful influence on the ratings for *intelligent*, *gallant*, *charming* and *sociable* for the older as compared to the younger group; this may reflect a level of experience that renders individuals more cautious towards “judging a book by its cover.” As to elegant diction, older participants gave even lower mean ratings on the scales for *long-winded*, *verbose*, *complicated*, und *stilted*, and an even higher mean rating for *concise* and on *elegant diction* (Wilk’s λ = 0.725, *F*(20,129) = 2.49, *p* < .01). That is, the pattern is the same, but it is more pronounced for the participants of higher age.

Younger and older participants also differed significantly in their ratings regarding how frequently individual items in the experimenter-selected object classes are elegant as well as to what degree they can be elegant (Wilk’s λ = 0.730, *F*(14,135) = 3.57, *p* < .001 and Wilk’s λ = 0.798, *F*(14,134) = 2.34, *p* < .01). Specifically, older participants were less upbeat regarding the elegance of hairstyles, underwear, furniture, and evening wear, and gave higher ratings for skyscrapers than the younger group.

In sum, the age-driven differences we found add nuances to our findings, but do not call into question the overall, multi-faceted and still very precise construct of elegance that emerges from our data.
