# Supplementary material for: Beauty, elegance, grace, and sexiness compared
Source: PLoS One. 2019 Jun 21;14(6):e0218728. doi: 10.1371/journal.pone.0218728 (PMC6588248; doi:10.1371/journal.pone.0218728)

### S5 Text. Bayes factor analyses for pair wise compassion of the four categories

We performed six pair-wise comparisons for all target categories computing Bayes factors [1-2] for each adjective pair using the ttestBF-function of the R-package BayesFactor [3] with the default settings in order to test on which of the 42 semantic differentials the categories are convergent and divergent.

In the following tables, the mean difference (2^nd^ column) and the log of the BF (3^rd^ column) for each adjective pair is listed, with color-coding for inconclusive or conclusive evidence for difference (log(BF) > 1) or no difference (log(BF) < −1).

| evidence for difference |
| --- |
| inconclusive |
| evidence for NO difference |

***References***

1. Kass RE, Raftery AE (1995) Bayes factors. Journal of the American Statistical Association 90: 773‒795. doi:10.1080/01621459.1995.10476572.

2. Morey RD, Romeijn J-W, Rouder JN (2016) The philosophy of Bayes factors and the quantification of statistical evidence. Journal of Mathematical Psychology 72: 6‒18. doi:10.1016/j.jmp.2015.11.001.

3. Morey RD, Rouder JN (2015) BayesFactor: Computation of Bayes Factors for Common Designs. 0.9.12-2 ed.

Beauty vs Elegance

| **adj.pairs** | **beauty - elegance** | **log(BF)** |
| --- | --- | --- |
| multi.colored -- monochrome | -1.22 | 14.24 |
| natural -- artificial | -0.86 | 6.98 |
| indulgent -- rigorous | -0.84 | 10.60 |
| democratic -- elitist | -0.82 | 7.24 |
| dreamy -- sober | -0.75 | 7.17 |
| emotional -- rational | -0.61 | 2.81 |
| down.to.earth -- divorced.from.reality | -0.58 | 2.22 |
| hot -- cool | -0.55 | 3.19 |
| young -- old | -0.38 | 0.94 |
| verbose -- concise | -0.33 | 0.09 |
| libidinous -- inhibited | -0.28 | -0.44 |
| pleasant -- unpleasant | -0.23 | -1.19 |
| simple -- baroque | -0.21 | -1.20 |
| reserved -- forceful | -0.17 | -1.48 |
| discreet -- richly.ornamented | -0.03 | -1.87 |
| soothing -- arousing | 0.01 | -1.90 |
| slender -- sturdy | 0.03 | -1.88 |
| educated -- uneducated | 0.04 | -1.87 |
| smart -- stupid | 0.07 | -1.80 |
| feminine -- masculine | 0.09 | -1.76 |
| extrovert -- introvert | 0.13 | -1.58 |
| harmonious -- disharmonious | 0.15 | -1.54 |
| witty -- inane | 0.16 | -1.43 |
| conspicuous -- inconspicuous | 0.27 | -0.94 |
| light -- heavy | 0.29 | -0.67 |
| delicate -- coarse | 0.31 | -0.44 |
| fragile -- robust | 0.32 | -0.66 |
| precious -- worthless | 0.32 | 0.12 |
| tasteful -- tasteless | 0.36 | 0.87 |
| tall -- short | 0.36 | 0.57 |
| fluent -- faltering | 0.54 | 3.32 |
| quiet -- loud | 0.55 | 3.23 |
| cultured -- uncultured | 0.58 | 4.41 |
| skillful -- clumsy | 0.61 | 4.65 |
| superior -- inferior | 0.61 | 6.19 |
| svelte -- bulky | 0.61 | 5.71 |
| rich -- poor | 0.62 | 4.64 |
| exceptional -- ordinary | 0.66 | 4.76 |
| expensive -- cheap | 0.68 | 6.23 |
| exquisite -- common | 0.68 | 5.25 |
| disciplined -- undisciplined | 0.71 | 6.53 |
| refined -- vulgar | 0.95 | 15.93 |


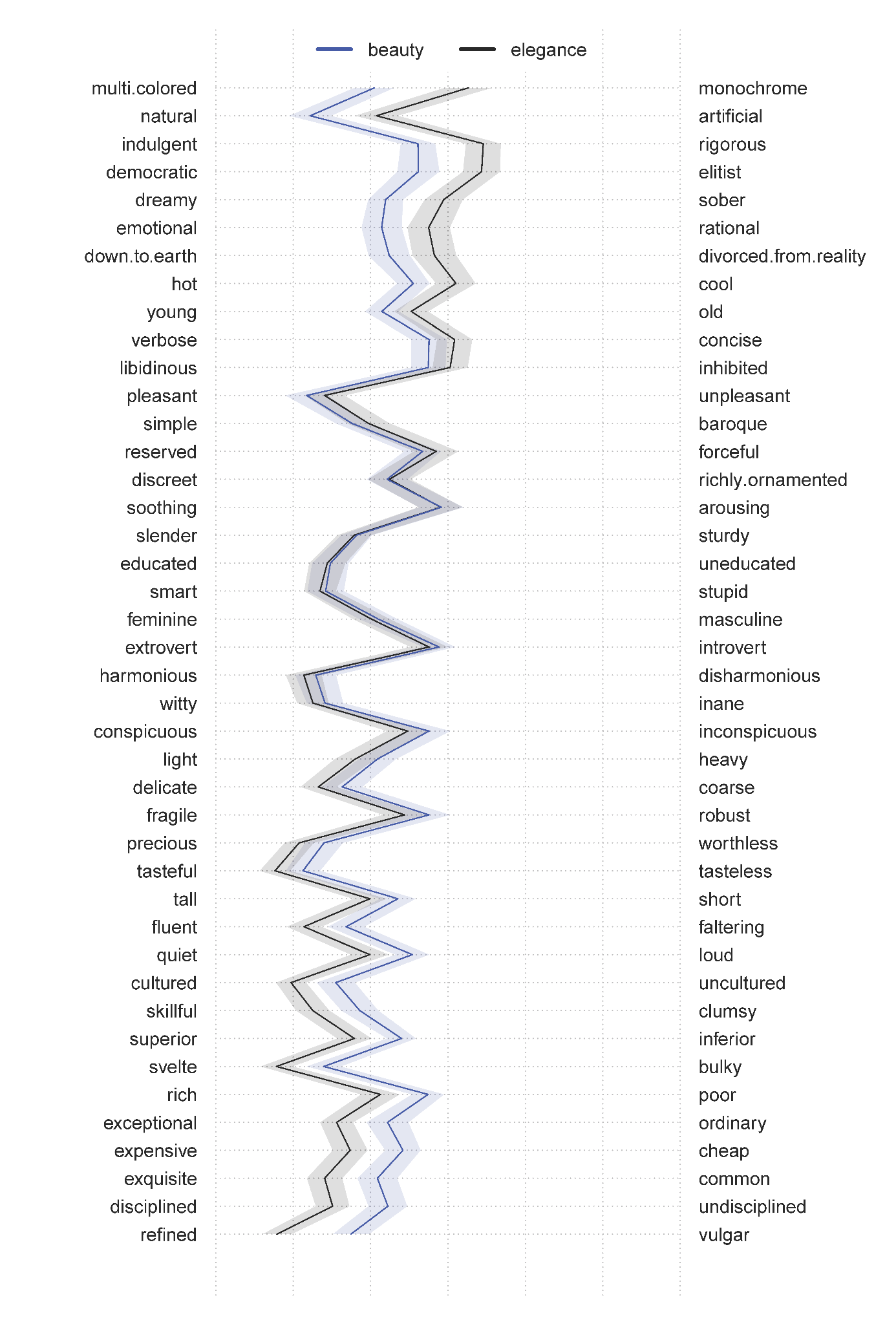


Beauty vs Grace

| **adj.pairs** | **beauty - grace** | **log(BF)** |
| --- | --- | --- |
| multi.colored -- monochrome | -0.93 | 9.34 |
| hot -- cool | -0.73 | 6.97 |
| democratic -- elitist | -0.70 | 3.73 |
| natural -- artificial | -0.57 | 1.55 |
| down.to.earth -- divorced.from.reality | -0.54 | 1.61 |
| emotional -- rational | -0.46 | 0.53 |
| young -- old | -0.41 | 1.41 |
| indulgent -- rigorous | -0.40 | 0.48 |
| libidinous -- inhibited | -0.24 | -0.98 |
| simple -- baroque | -0.23 | -1.04 |
| pleasant -- unpleasant | -0.20 | -1.40 |
| dreamy -- sober | -0.18 | -1.42 |
| verbose -- concise | -0.16 | -1.44 |
| slender -- sturdy | -0.11 | -1.67 |
| feminine -- masculine | -0.09 | -1.76 |
| discreet -- richly.ornamented | -0.06 | -1.82 |
| educated -- uneducated | -0.06 | -1.82 |
| tasteful -- tasteless | -0.06 | -1.81 |
| witty -- inane | -0.05 | -1.83 |
| smart -- stupid | -0.01 | -1.87 |
| delicate -- coarse | 0.02 | -1.88 |
| fragile -- robust | 0.03 | -1.85 |
| extrovert -- introvert | 0.07 | -1.80 |
| tall -- short | 0.10 | -1.71 |
| precious -- worthless | 0.13 | -1.61 |
| conspicuous -- inconspicuous | 0.15 | -1.63 |
| reserved -- forceful | 0.18 | -1.44 |
| light -- heavy | 0.26 | -0.95 |
| harmonious -- disharmonious | 0.28 | -0.80 |
| soothing -- arousing | 0.28 | -1.02 |
| expensive -- cheap | 0.29 | -0.26 |
| svelte -- bulky | 0.35 | 0.01 |
| superior -- inferior | 0.36 | 0.35 |
| fluent -- faltering | 0.40 | 0.16 |
| rich -- poor | 0.41 | 1.53 |
| cultured -- uncultured | 0.46 | 1.14 |
| skillful -- clumsy | 0.51 | 2.25 |
| quiet -- loud | 0.60 | 3.24 |
| exquisite -- common | 0.61 | 3.36 |
| disciplined -- undisciplined | 0.63 | 3.05 |
| refined -- vulgar | 0.69 | 5.01 |
| exceptional -- ordinary | 0.77 | 6.71 |


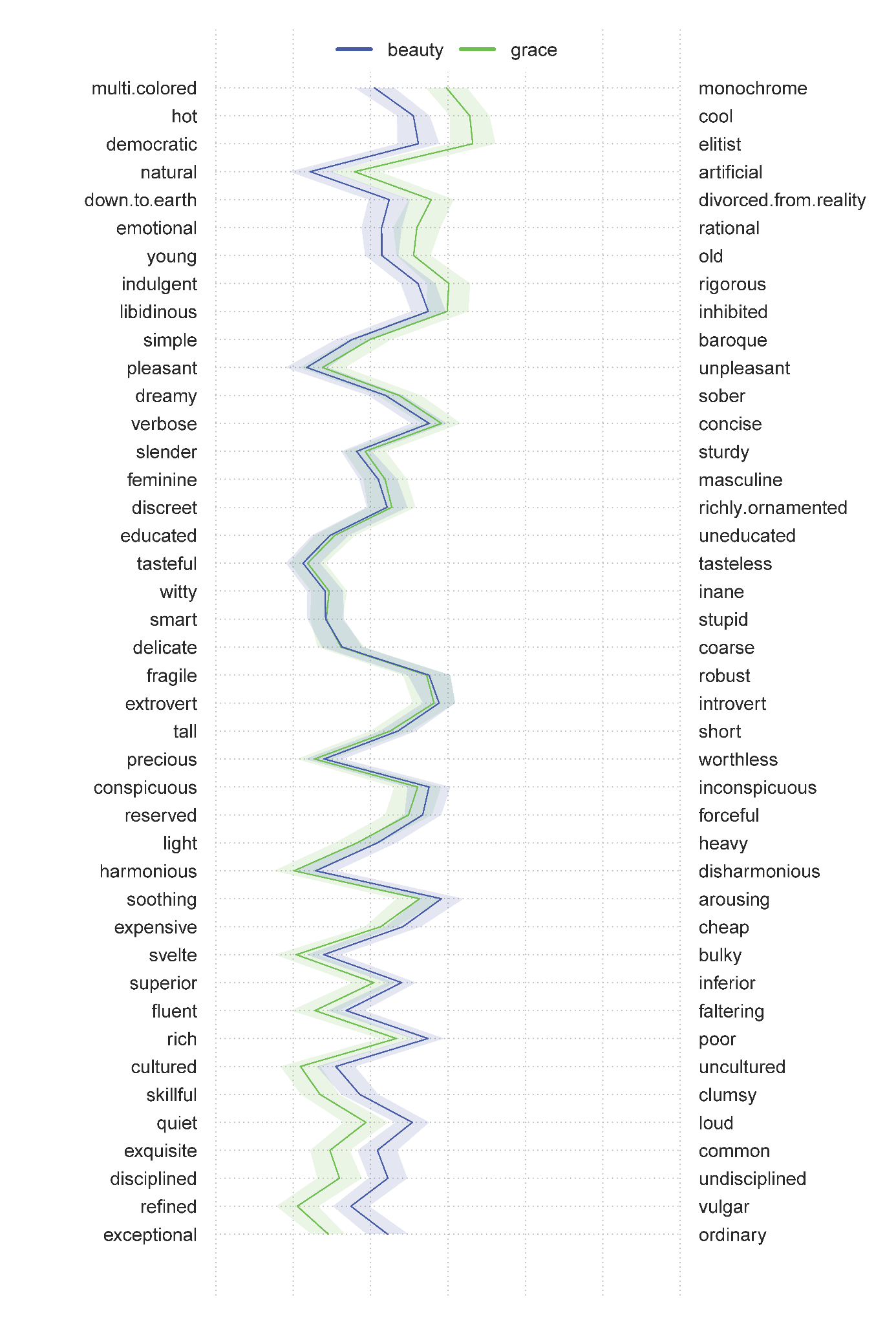


Beauty vs Sexiness

| **adj.pairs** | **beauty - sexiness** | **log(BF)** |
| --- | --- | --- |
| soothing -- arousing | -1.38 | 16.28 |
| delicate -- coarse | -0.97 | 9.44 |
| fragile -- robust | -0.93 | 9.17 |
| feminine -- masculine | -0.86 | 4.54 |
| simple -- baroque | -0.76 | 7.02 |
| reserved -- forceful | -0.76 | 7.18 |
| quiet -- loud | -0.74 | 7.46 |
| refined -- vulgar | -0.64 | 4.10 |
| discreet -- richly.ornamented | -0.63 | 3.37 |
| indulgent -- rigorous | -0.35 | 0.36 |
| harmonious -- disharmonious | -0.30 | -0.75 |
| natural -- artificial | -0.29 | -0.82 |
| tasteful -- tasteless | -0.29 | -0.73 |
| dreamy -- sober | -0.24 | -0.91 |
| expensive -- cheap | -0.23 | -0.84 |
| pleasant -- unpleasant | -0.23 | -1.23 |
| svelte -- bulky | -0.21 | -1.19 |
| precious -- worthless | -0.20 | -1.27 |
| verbose -- concise | -0.16 | -1.36 |
| fluent -- faltering | -0.13 | -1.62 |
| disciplined -- undisciplined | -0.12 | -1.69 |
| emotional -- rational | -0.07 | -1.79 |
| cultured -- uncultured | -0.06 | -1.84 |
| light -- heavy | -0.05 | -1.83 |
| witty -- inane | -0.05 | -1.87 |
| down.to.earth -- divorced.from.reality | -0.04 | -1.88 |
| democratic -- elitist | -0.01 | -1.87 |
| rich -- poor | 0.04 | -1.85 |
| exquisite -- common | 0.04 | -1.86 |
| slender -- sturdy | 0.07 | -1.77 |
| superior -- inferior | 0.08 | -1.76 |
| multi.colored -- monochrome | 0.08 | -1.81 |
| educated -- uneducated | 0.10 | -1.76 |
| young -- old | 0.13 | -1.59 |
| smart -- stupid | 0.17 | -1.42 |
| exceptional -- ordinary | 0.19 | -1.48 |
| skillful -- clumsy | 0.22 | -1.11 |
| tall -- short | 0.32 | -0.25 |
| conspicuous -- inconspicuous | 0.37 | -0.23 |
| libidinous -- inhibited | 0.74 | 6.98 |
| extrovert -- introvert | 0.95 | 14.31 |
| hot -- cool | 0.97 | 11.85 |


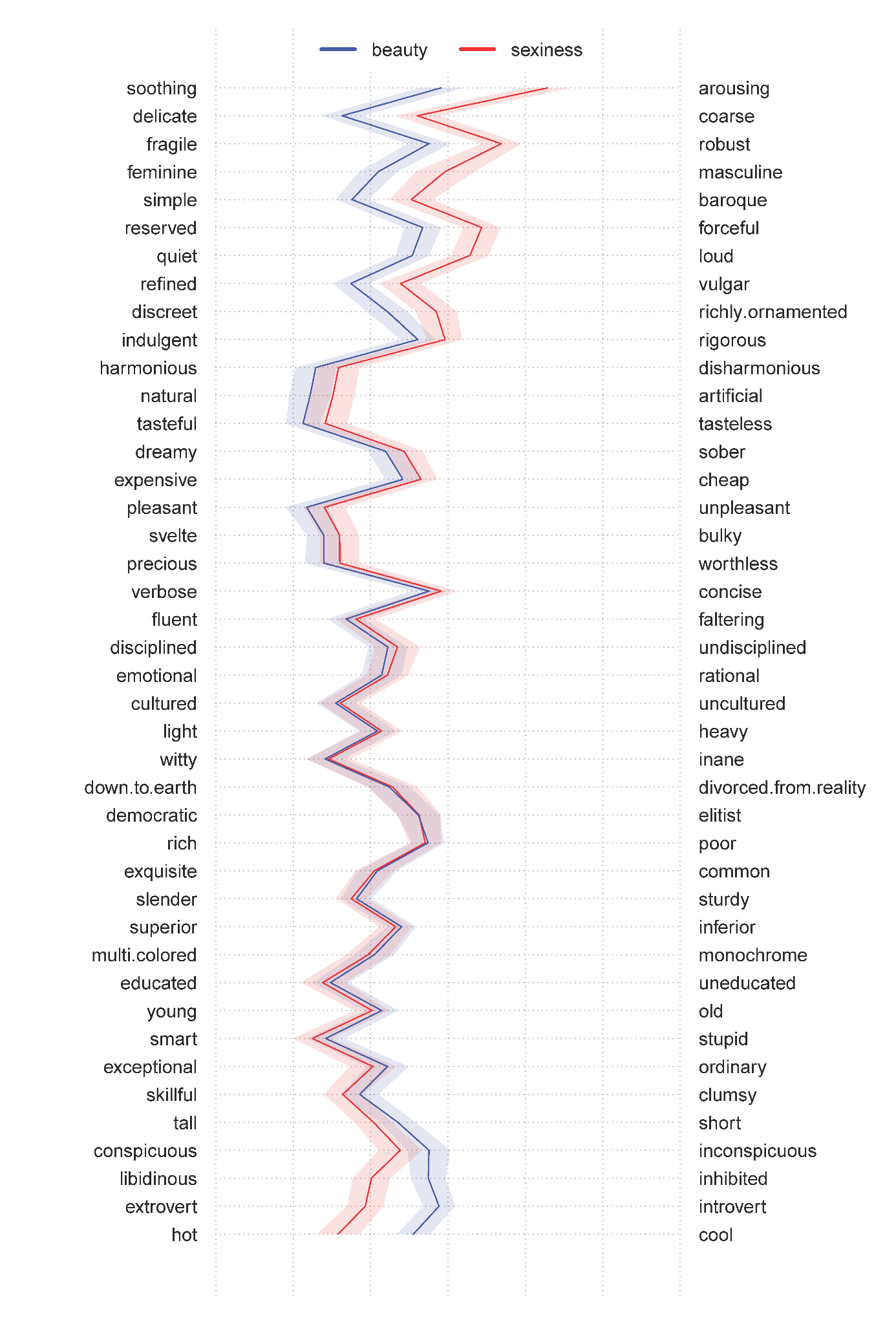


Elegance vs Grace

| **adj.pairs** | **elegance - grace** | **log(BF)** |
| --- | --- | --- |
| tasteful -- tasteless | -0.42 | 1.67 |
| expensive -- cheap | -0.39 | 1.19 |
| delicate -- coarse | -0.29 | -0.77 |
| fragile -- robust | -0.29 | -0.98 |
| tall -- short | -0.26 | -0.72 |
| refined -- vulgar | -0.26 | -0.67 |
| svelte -- bulky | -0.26 | -0.82 |
| superior -- inferior | -0.25 | -0.93 |
| witty -- inane | -0.21 | -1.02 |
| rich -- poor | -0.21 | -1.16 |
| precious -- worthless | -0.20 | -1.04 |
| hot -- cool | -0.18 | -1.42 |
| feminine -- masculine | -0.18 | -1.44 |
| fluent -- faltering | -0.14 | -1.61 |
| slender -- sturdy | -0.14 | -1.57 |
| conspicuous -- inconspicuous | -0.13 | -1.68 |
| cultured -- uncultured | -0.12 | -1.61 |
| educated -- uneducated | -0.10 | -1.73 |
| skillful -- clumsy | -0.09 | -1.73 |
| disciplined -- undisciplined | -0.09 | -1.77 |
| smart -- stupid | -0.08 | -1.73 |
| exquisite -- common | -0.07 | -1.80 |
| extrovert -- introvert | -0.06 | -1.81 |
| young -- old | -0.03 | -1.86 |
| discreet -- richly.ornamented | -0.03 | -1.86 |
| light -- heavy | -0.02 | -1.86 |
| simple -- baroque | -0.02 | -1.88 |
| pleasant -- unpleasant | 0.03 | -1.87 |
| libidinous -- inhibited | 0.04 | -1.86 |
| down.to.earth -- divorced.from.reality | 0.04 | -1.86 |
| quiet -- loud | 0.05 | -1.84 |
| exceptional -- ordinary | 0.11 | -1.65 |
| democratic -- elitist | 0.11 | -1.71 |
| harmonious -- disharmonious | 0.13 | -1.63 |
| emotional -- rational | 0.15 | -1.62 |
| verbose -- concise | 0.17 | -1.37 |
| soothing -- arousing | 0.27 | -1.07 |
| natural -- artificial | 0.28 | -1.05 |
| multi.colored -- monochrome | 0.28 | -0.97 |
| reserved -- forceful | 0.36 | -0.50 |
| indulgent -- rigorous | 0.45 | 1.03 |
| dreamy -- sober | 0.57 | 2.48 |


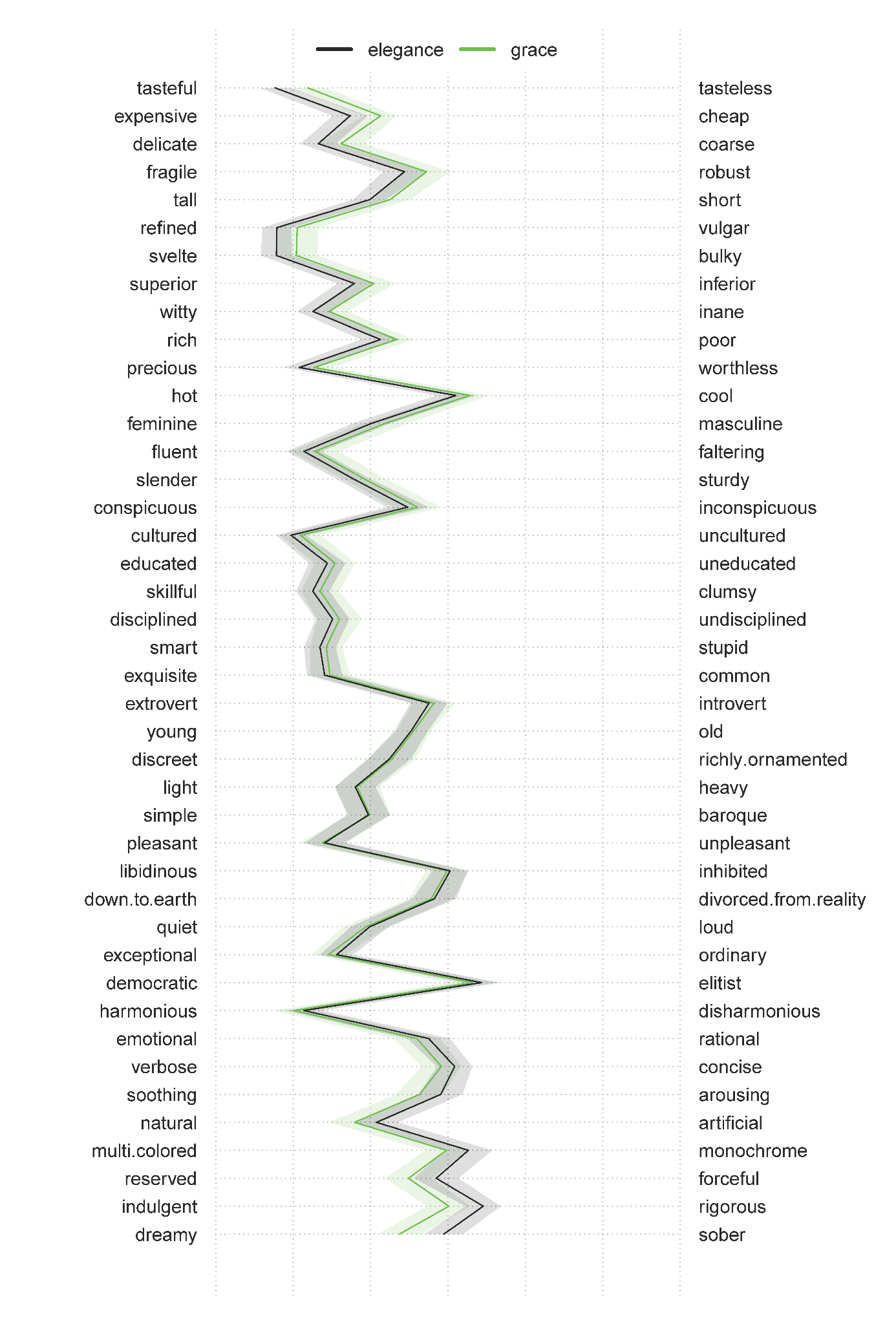


Elegance vs Sexiness

| **adj.pairs** | **elegance - sexiness** | **log(BF)** |
| --- | --- | --- |
| refined -- vulgar | -1.60 | 36.52 |
| soothing -- arousing | -1.38 | 16.70 |
| quiet -- loud | -1.29 | 21.93 |
| delicate -- coarse | -1.28 | 19.18 |
| fragile -- robust | -1.25 | 17.08 |
| feminine -- masculine | -0.95 | 6.21 |
| expensive -- cheap | -0.91 | 13.58 |
| disciplined -- undisciplined | -0.84 | 7.85 |
| svelte -- bulky | -0.82 | 9.47 |
| fluent -- faltering | -0.67 | 5.39 |
| tasteful -- tasteless | -0.65 | 4.47 |
| exquisite -- common | -0.64 | 4.80 |
| cultured -- uncultured | -0.63 | 5.05 |
| discreet -- richly.ornamented | -0.60 | 2.67 |
| reserved -- forceful | -0.59 | 2.61 |
| rich -- poor | -0.58 | 4.01 |
| simple -- baroque | -0.55 | 1.87 |
| superior -- inferior | -0.53 | 3.72 |
| precious -- worthless | -0.53 | 3.14 |
| exceptional -- ordinary | -0.47 | 1.09 |
| harmonious -- disharmonious | -0.45 | 1.04 |
| skillful -- clumsy | -0.39 | 0.73 |
| light -- heavy | -0.34 | -0.35 |
| witty -- inane | -0.20 | -1.27 |
| tall -- short | -0.05 | -1.86 |
| pleasant -- unpleasant | 0.00 | -1.89 |
| slender -- sturdy | 0.04 | -1.86 |
| educated -- uneducated | 0.06 | -1.85 |
| conspicuous -- inconspicuous | 0.10 | -1.77 |
| smart -- stupid | 0.10 | -1.72 |
| verbose -- concise | 0.17 | -1.23 |
| indulgent -- rigorous | 0.50 | 2.53 |
| young -- old | 0.50 | 2.81 |
| dreamy -- sober | 0.51 | 2.07 |
| emotional -- rational | 0.53 | 1.64 |
| down.to.earth -- divorced.from.reality | 0.54 | 1.03 |
| natural -- artificial | 0.56 | 1.87 |
| democratic -- elitist | 0.81 | 6.68 |
| extrovert -- introvert | 0.82 | 8.88 |
| libidinous -- inhibited | 1.02 | 14.41 |
| multi.colored -- monochrome | 1.29 | 14.89 |
| hot -- cool | 1.52 | 25.44 |


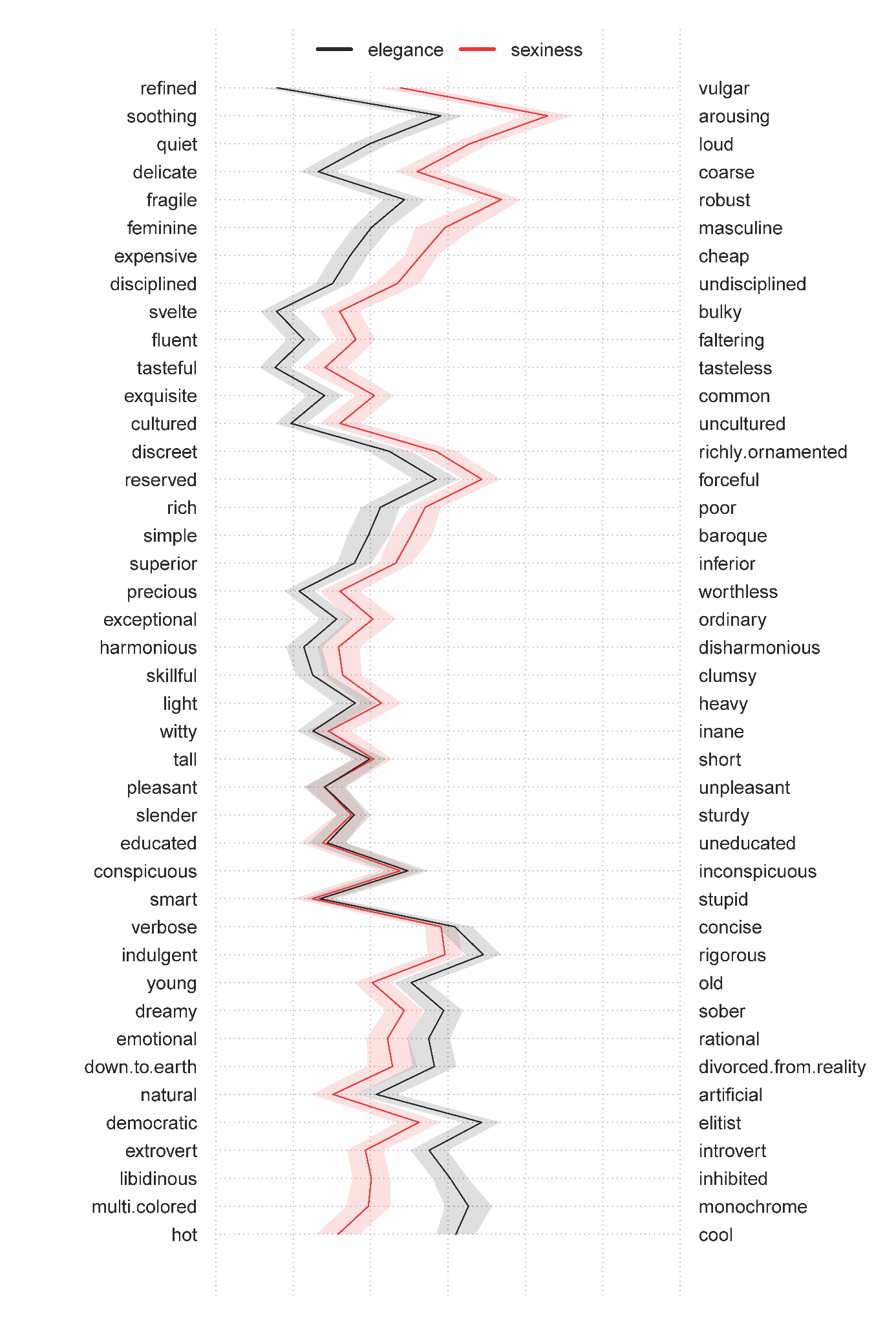


Grace vs Sexiness

| **adj.pairs** | **grace - sexiness** | **log(BF)** |
| --- | --- | --- |
| soothing -- arousing | -1.66 | 22.48 |
| quiet -- loud | -1.34 | 20.20 |
| refined -- vulgar | -1.33 | 19.18 |
| delicate -- coarse | -0.99 | 8.24 |
| fragile -- robust | -0.97 | 8.66 |
| reserved -- forceful | -0.95 | 8.83 |
| feminine -- masculine | -0.77 | 2.84 |
| disciplined -- undisciplined | -0.75 | 4.19 |
| harmonious -- disharmonious | -0.58 | 2.45 |
| exceptional -- ordinary | -0.58 | 2.41 |
| discreet -- richly.ornamented | -0.57 | 1.83 |
| exquisite -- common | -0.57 | 2.96 |
| svelte -- bulky | -0.56 | 2.24 |
| simple -- baroque | -0.53 | 1.63 |
| fluent -- faltering | -0.53 | 1.41 |
| expensive -- cheap | -0.52 | 3.91 |
| cultured -- uncultured | -0.51 | 1.66 |
| rich -- poor | -0.37 | 1.01 |
| precious -- worthless | -0.33 | -0.16 |
| light -- heavy | -0.31 | -0.67 |
| skillful -- clumsy | -0.29 | -0.55 |
| superior -- inferior | -0.28 | -0.61 |
| tasteful -- tasteless | -0.23 | -1.18 |
| dreamy -- sober | -0.06 | -1.81 |
| pleasant -- unpleasant | -0.02 | -1.86 |
| verbose -- concise | 0.00 | -1.83 |
| witty -- inane | 0.01 | -1.88 |
| indulgent -- rigorous | 0.05 | -1.83 |
| educated -- uneducated | 0.16 | -1.56 |
| slender -- sturdy | 0.18 | -1.33 |
| smart -- stupid | 0.18 | -1.30 |
| tall -- short | 0.21 | -1.19 |
| conspicuous -- inconspicuous | 0.23 | -1.32 |
| natural -- artificial | 0.28 | -1.08 |
| emotional -- rational | 0.38 | -0.23 |
| down.to.earth -- divorced.from.reality | 0.50 | 0.53 |
| young -- old | 0.54 | 3.34 |
| democratic -- elitist | 0.69 | 3.37 |
| extrovert -- introvert | 0.89 | 9.01 |
| libidinous -- inhibited | 0.98 | 10.79 |
| multi.colored -- monochrome | 1.01 | 9.90 |
| hot -- cool | 1.70 | 30.52 |


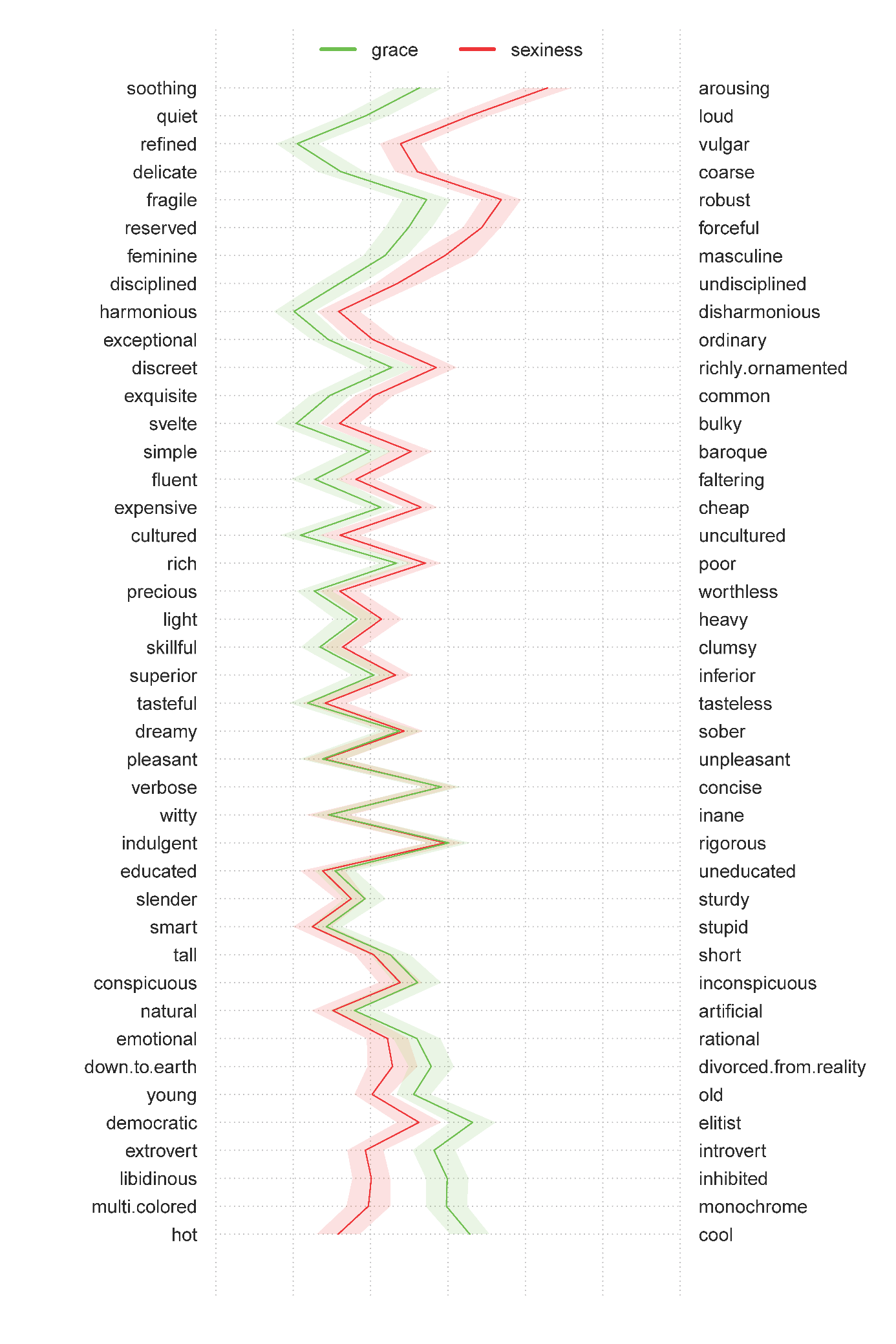

Supplement: S5 Text — (DOCX) [file pone.0218728.s005.docx]
