# Supplementary material for: Beauty, elegance, grace, and sexiness compared
Source: PLoS One. 2019 Jun 21;14(6):e0218728. doi: 10.1371/journal.pone.0218728 (PMC6588248; doi:10.1371/journal.pone.0218728)

### S6 Text. Analysis of the semantic differential ratings for beauty, elegance, grace, and sexiness by gender

The profiles for the concept of **beauty** did not differ significantly between men and women (n=48 and n=60, respectively; Wilk’s λ = 0.35, Pillai’s trace = 0.65, *F*(42,32) = 1.43, *p* = .15). Univariate ANOVAs likewise revealed no significant differences (false-detection rate corrected).


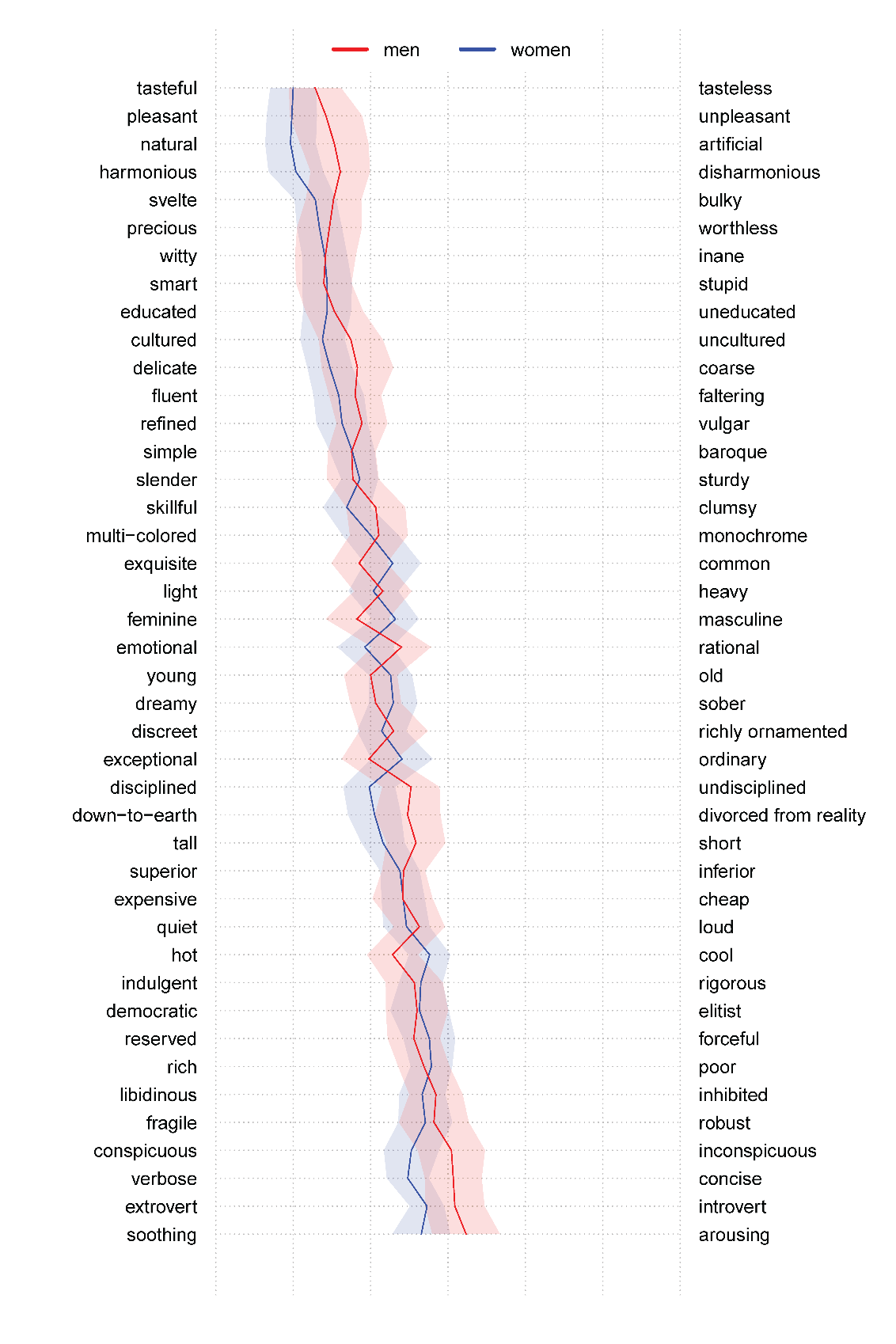


The profiles for the concept of **elegance** did not differ significantly between men and women (n=45 and n=66, respectively; Wilk’s λ = 0.43, Pillai’s trace = 0.57, *F*(42,35) = 1.10, *p* = .39). Univariate ANOVAs likewise revealed no significant differences (false-detection rate corrected).


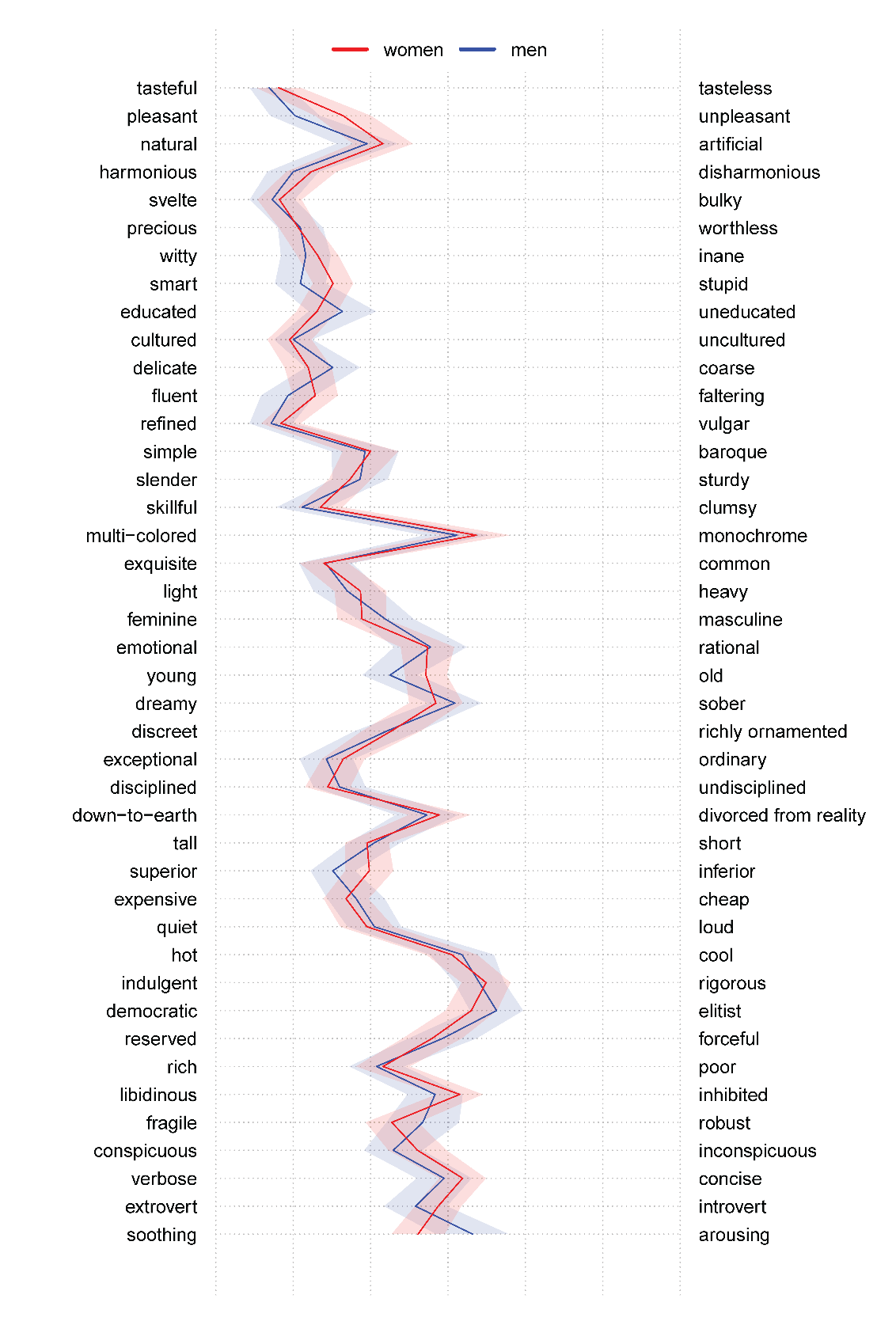


The profiles for the concept of **grace** did not differ significantly between men and women (n=46 and n=56, respectively; Wilk’s λ = 0.53, Pillai’s trace = 0.47, *F*(42,31) = 0.66, *p* = .89). Univariate ANOVAs likewise revealed no significant differences (false-detection rate corrected).


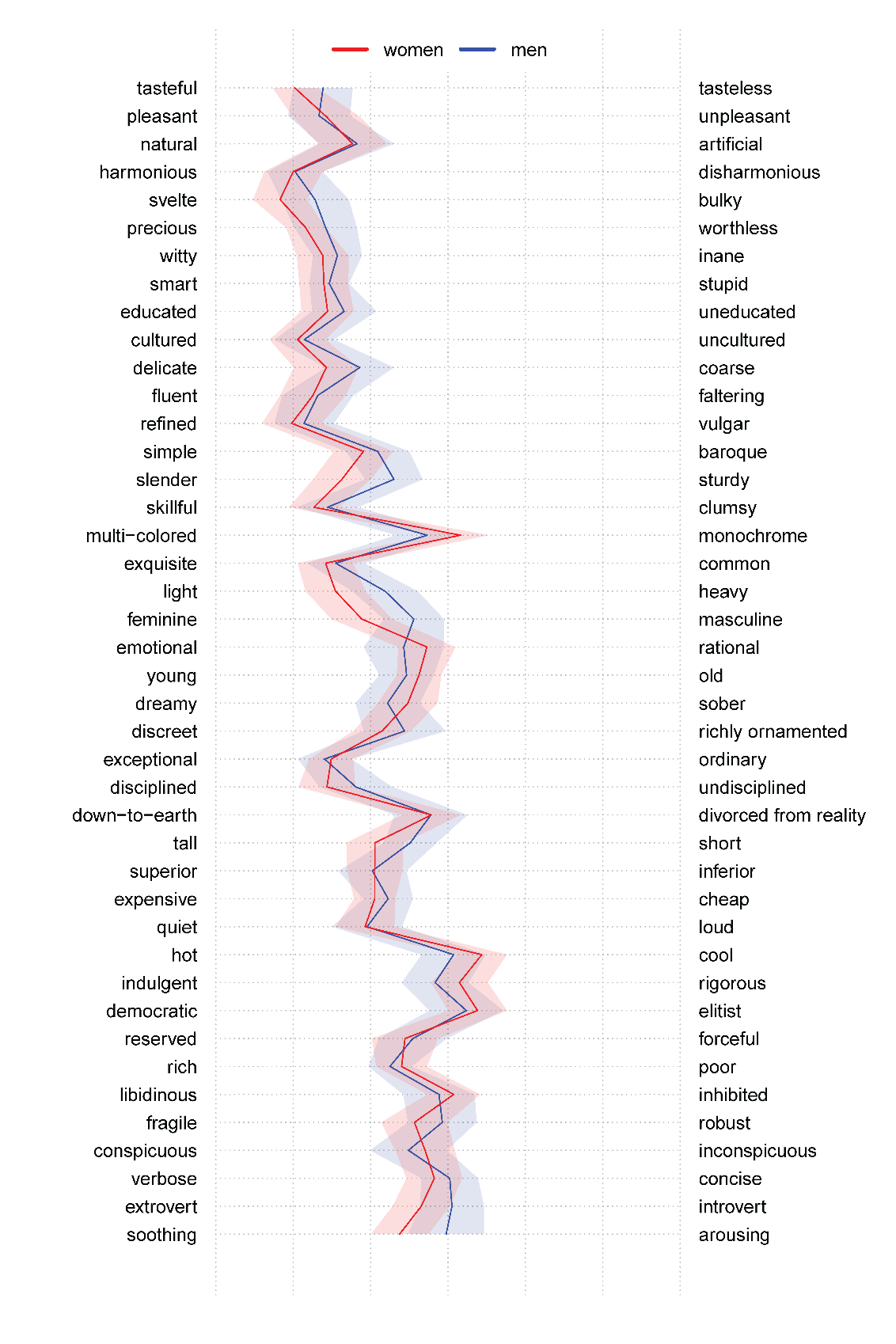


The profiles for the concept of **sexiness** differed significantly between men and women (n=42 and n=66, respectively; Wilk’s λ = 0.21, Pillai’s trace = 0.79, *F*(42,30) = 2.64, *p* = .003). Univariate ANOVAs (fdr corrected) revealed significant differences for *feminine_masculine*, with men rating sexiness as more *feminine* and women as more *masculine*, *F*(1,71) = 19.95, *p* < .001), and for *tall-short*, with women associating sexiness with greater body height (*tall*) than men, *F*(1,71) = 12.29, *p* = .004).


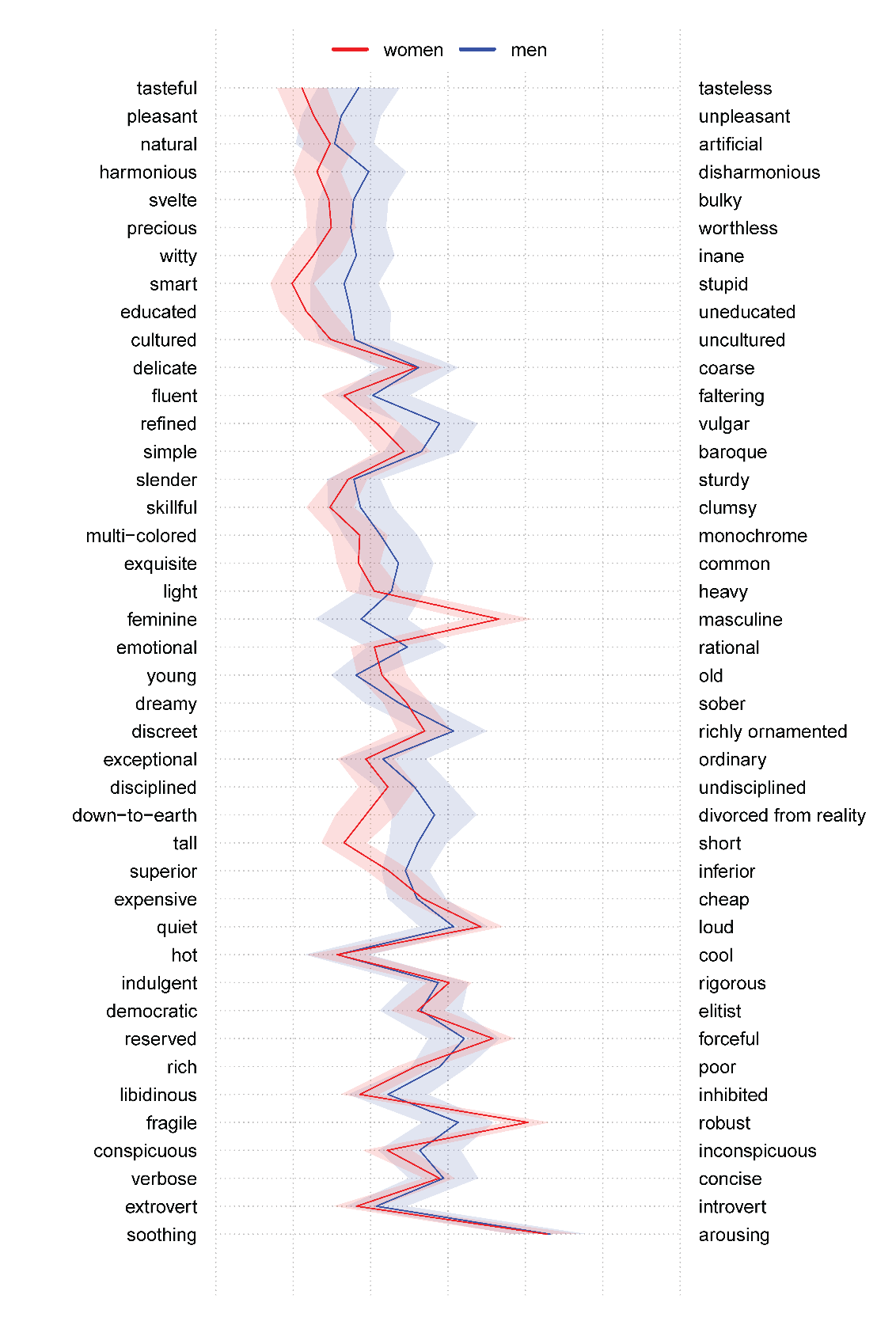

Supplement: S6 Text — (DOCX) [file pone.0218728.s006.docx]
