# Supplementary figures and images for: Beauty, elegance, grace, and sexiness compared
Source: PLoS One. 2019 Jun 21;14(6):e0218728. doi: 10.1371/journal.pone.0218728 (PMC6588248; doi:10.1371/journal.pone.0218728)

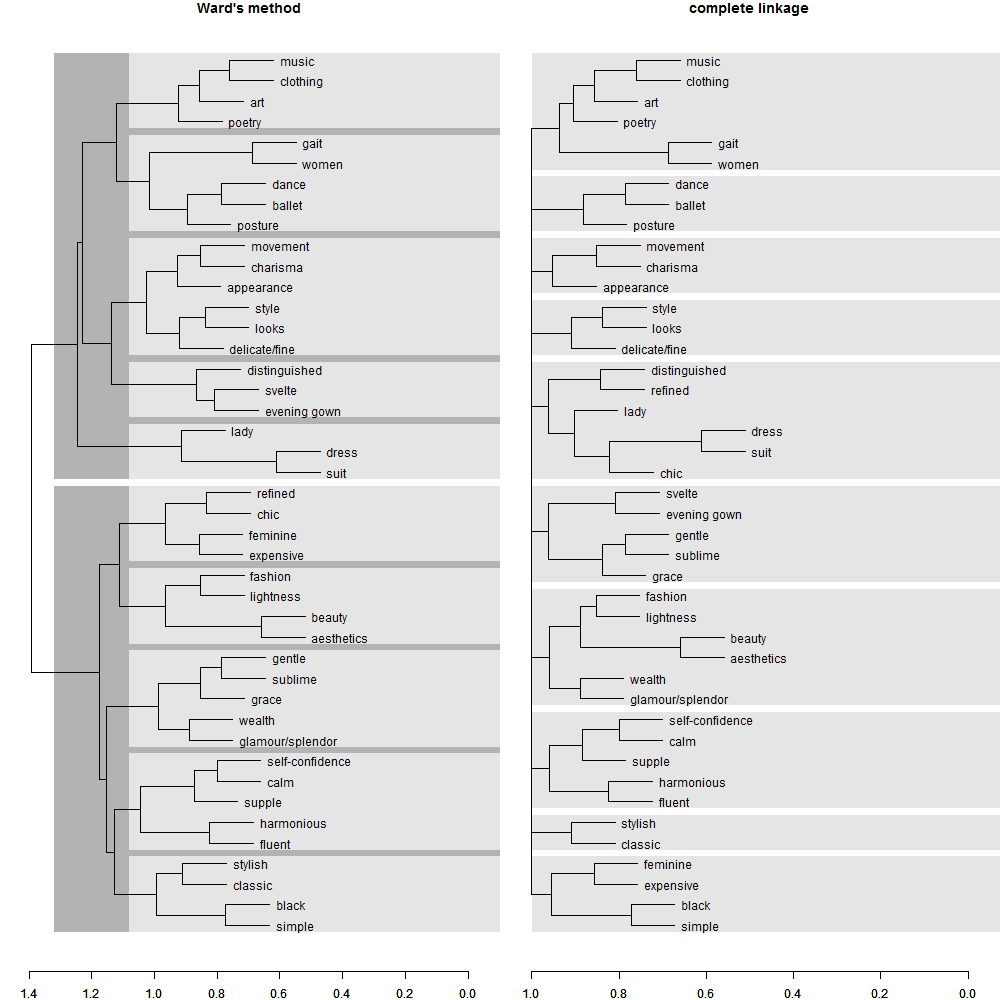

Supplement: S1 Fig — (PNG) [file pone.0218728.s007.png]

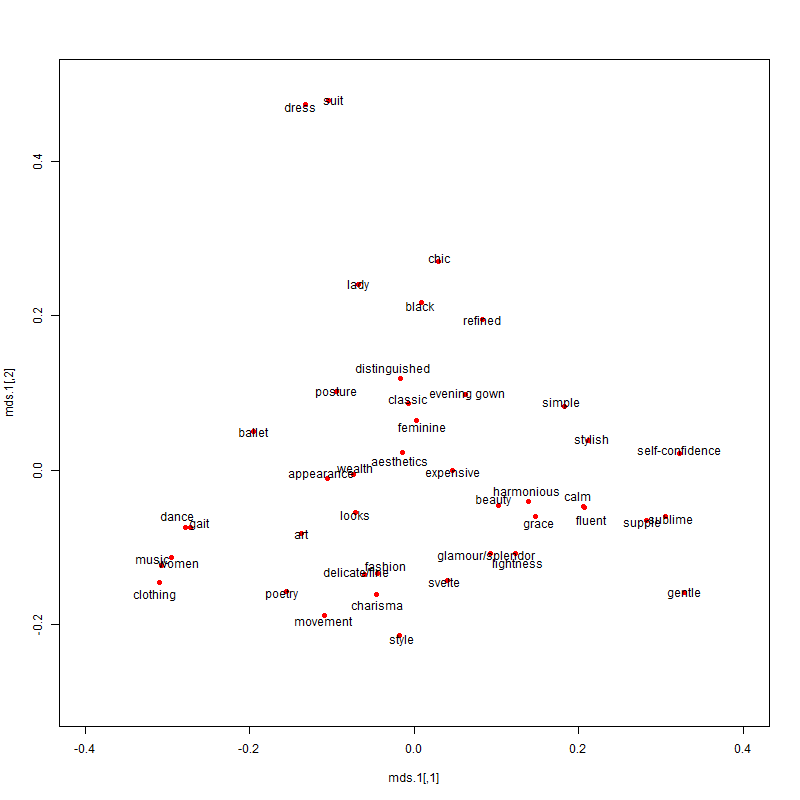

Supplement: S2 Fig — (PNG) [file pone.0218728.s008.png]

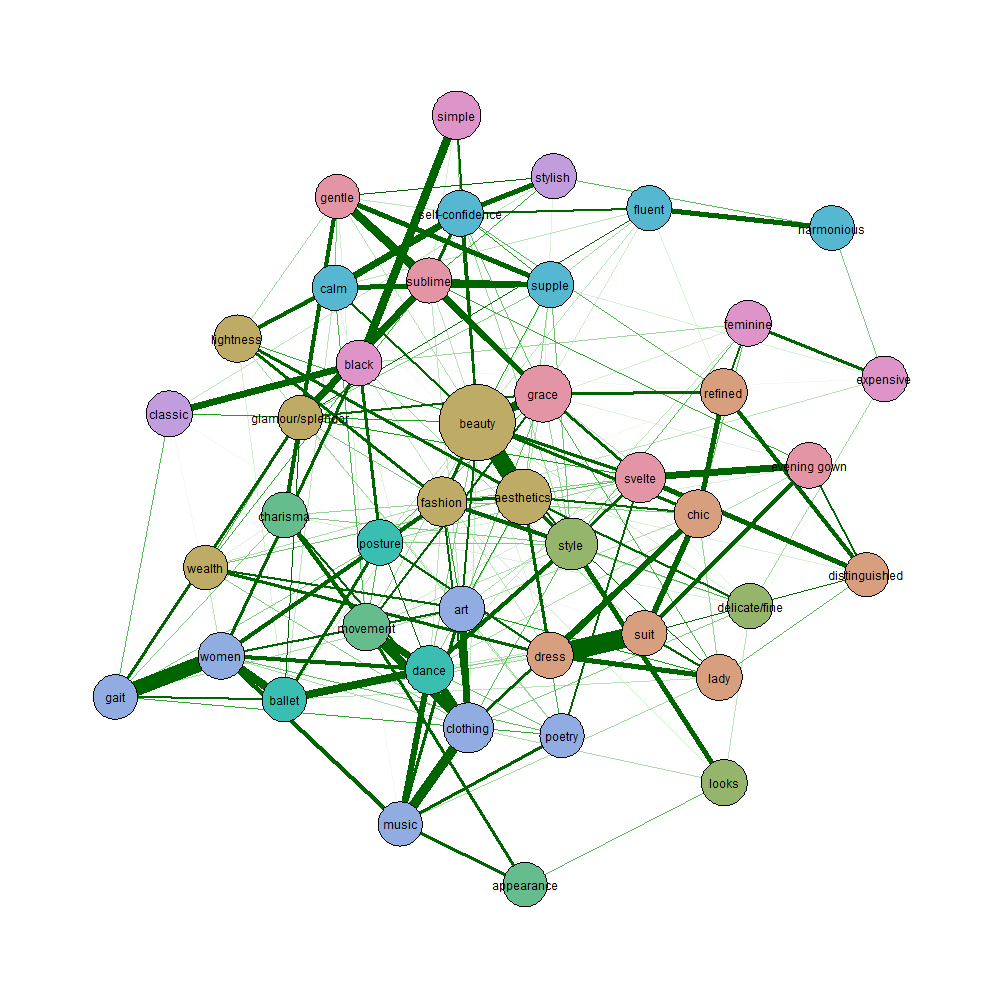

Supplement: S3 Fig — (PNG) [file pone.0218728.s009.png]

**Aspects of build**  
**(sorted by mean of men & women)**

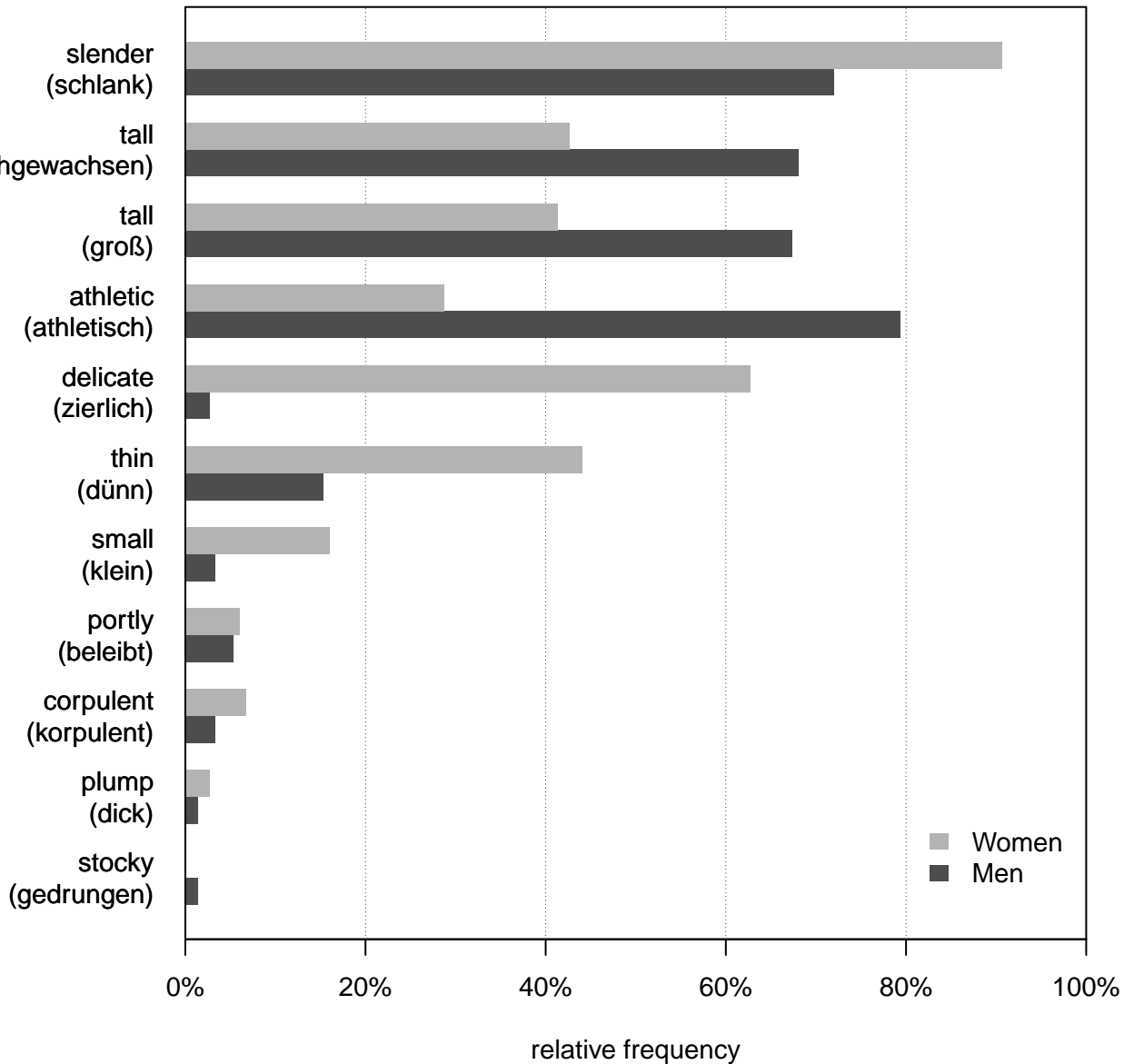

Supplement: S4 Fig — (PDF) [file pone.0218728.s010.pdf]

# body size

small

medium

tall

Women

Men

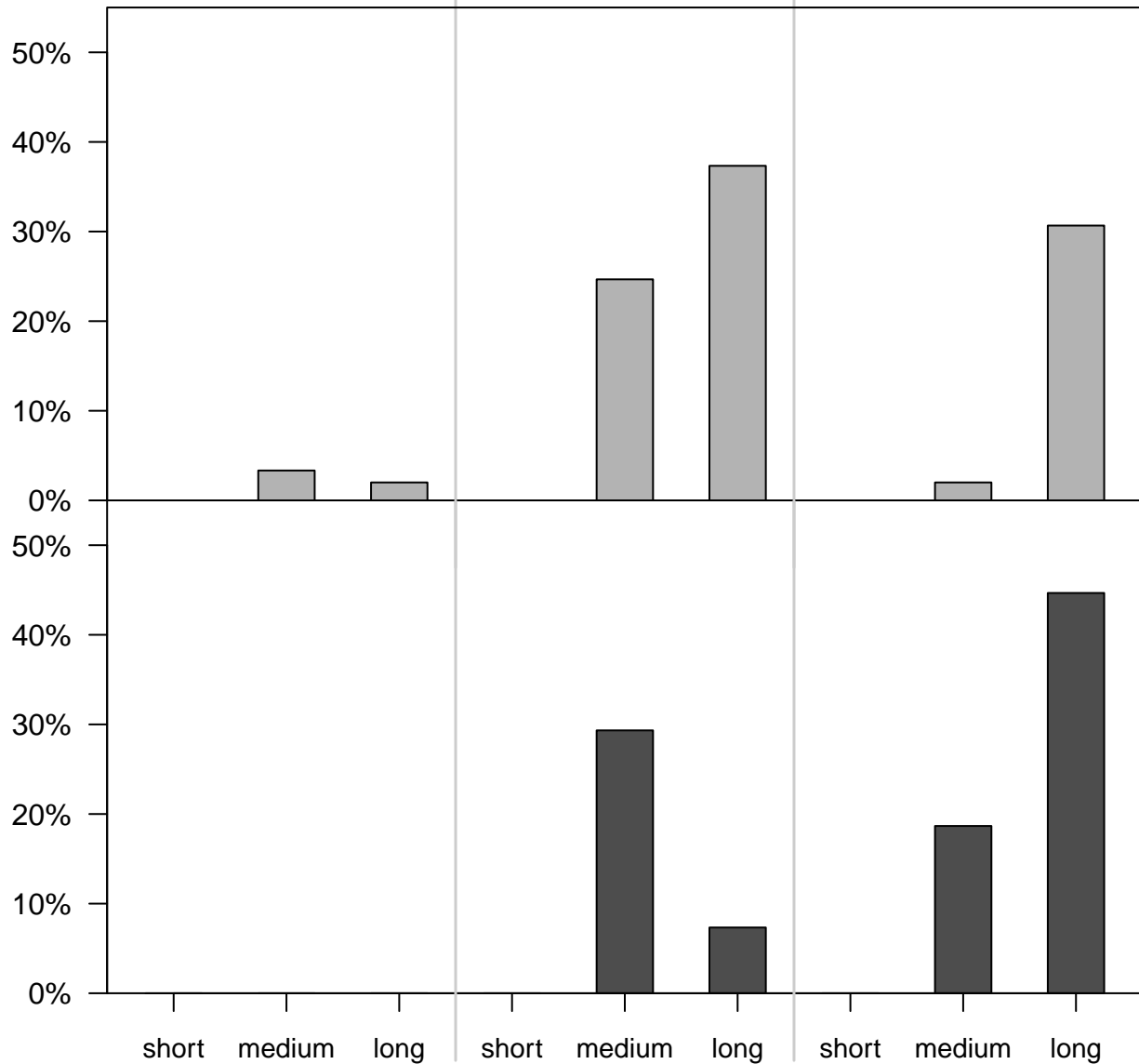

leg length

Supplement: S5 Fig — (PDF) [file pone.0218728.s011.pdf]
